# Antibacterial and Antibiofilm Activity of Titanium Treated with Hybrid Phospholipid Films Containing Carbonate Hydroxyapatite and Silver Nanoparticles

**DOI:** 10.1021/acsomega.5c09762

**Published:** 2026-02-26

**Authors:** Carla Roberta de Oliveira Maciel, Ailton Cravo Moraes Filho, Antonieta Catalina Varela Garcia, Viviane de Cássia Oliveira, Ana Paula Ramos, Ricardo Faria Ribeiro, Marcelle Beathriz Fernandes da Silva, Rafael Soares Stenico, Marcia Andreia Mesquita Silva da Veiga, Cássio do Nascimento

**Affiliations:** † Ribeirão Preto School of Dentistry, São Paulo University (USP), Dental Materials and Prosthodontics, Ribeirão Preto, São Paulo, 14040-904, Brazil; ‡ University of Sao Paulo, Department of Chemistry-FFCLRP-USP Av, Bandeirantes, 3900, Ribeirao Preto, São Paulo, 14040-901, Brazil; § Bone Research Lab, Ribeirão Preto School of Dentistry, University of São Paulo, Ribeirão Preto, São Paulo, 14040-904, Brazil

## Abstract

Bacterial contamination and low osteogenic activity are the major causes of dental implant failure. The development of titanium coatings has provided new research directions to improve both antibacterial and osteogenic activity. In this study, we constructed phospholipidic films containing carbonate hydroxyapatite added or not to silver nanoparticles. The purpose was to reduce the biofilm formation while maintaining the osteogenic potential of surfaces. Phospholipid monolayers were transferred to titanium by Langmuir–Blodgett (LB) technique and resulted in dense, ordered and uniform films. Modified surfaces were evaluated by X-ray photoelectron spectroscopy, energy-dispersive spectroscopy, Fourier-transform infrared spectroscopy, and atomic force microscopy to confirm that films were successfully coated onto the titanium substrate. In addition, surface free energy and roughness analyses indicated that surfaces were smooth and hydrophilic. ICP-OES analysis confirmed the absence of silver nanoparticle lixiviation from the coatings. Furthermore, the coatings had great biocompatibility and promoted the proliferation of osteoblast-like cells. Microbiological findings showed that biofilm formation and bacterial adhesion were significantly reduced for the experimental coatings; species closely related to peri-implant diseases and associated with increased biofilm volume were shown reduced. Addition of silver nanoparticles did not improve biofilm control. In conclusion, the phospholipidic films proposed to modify the titanium surfaces are beneficial for osseointegration and can act as a promising method to reduce the biofilm formation and bacterial colonization for dental implants.

## Graphical abstract

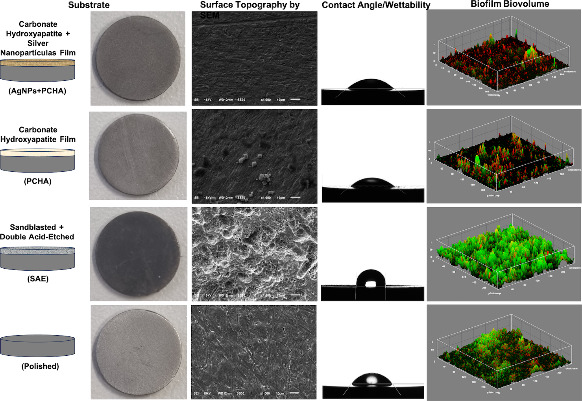

## 1 Introduction

The number of patients using dental implants has substantially increased over the last few decades. Implant-supported restorations have become an increasingly treatment choice for replacing missing teeth, mainly in older adults.^1,2^ This might be explained in part by the life expectancy increasing around the world and the scientific advances in the field of implant dentistry. Advances in tissue engineering, development of new materials and new technologies, including methods for modifying the surface of materials, have boosted the use of dental implants.^3^ Commercially pure titanium remains the preferred biomaterial for dental implants; the main reasons for the choice are the high degree of biocompatibility, resistance to corrosion and good mechanical properties.^4^

The success of dental implants is primary related to their primary stability and successful osseointegration.^5^ In this context, several approaches have focused on the treatment of titanium aiming to enhance the bioactivity of the implant surface and to promote or facilitate the osseointegration process.^6^ Indeed, literature reports high cumulative implant and prosthetic survival rates; percentage values reach 95% considering single-unit, partial or complete structures even up to 20 years of follow-up.^7,8^

Despite the safety and predictability of implant-supported fixed dental prosthesis, complications may occur and result in implant failure. Late failure is more likely to be caused by microbial infection that compromises the success of osseointegration process.^9^ The bacterial adhesion and proliferation on dental implant surfaces represent a significant clinical challenge and remain as a major concern in the long term success of implant-supported restorations, irrespectively the major advances in tissue engineering and surface treatments. Bacteria colonize around the implant immediately after implantation in the oral cavity and form complex biofilms.^10^ Biofilm disruption may cause microbial infections and lead to inflammatory conditions affecting both soft tissue and supporting bone, which might ultimately result in the implant loss.^11^

Controlling oral hygiene and removing the biofilm associated with peri-implant infections can potentially reverse the inflammation process. However, the complex architecture of biofilm containing different bacterial clusters associated with the surface topographic characteristics of implants makes this process quite difficult.^12^ Thus, focusing on the new strategies for the prevention of bacterial adhesion and colonization of dental implants may effectively contribute to reduce the implant-related infections and disease progression. Recently, surface modifications of titanium using biocompatible coatings have been proposed to improve its antimicrobial features while maintaining the bioactivity to the bone cells.^13^

Silver nanoparticles (AgNPs) are one of the most widely used antimicrobial agent due to its broad-spectrum antimicrobial activity, active against multiple pathogen types with efficient action mechanisms against bacteria and fungi.^14^ In addition, they have been linked to giving cells resistance to oxidative stress, which has been related to the development and progression of periodontal disease.^15^ AgNPs-based film coatings have showed an efficient antimicrobial effect on inhibiting both bacteria and fungi proliferation in the oral microbiota. The efficiency of AgNPs activity is mainly related to the high contact surface area provided by the uniform distribution of homogeneous small size particles.^16^ However, literature shows that nanoparticles tend to aggregate, which might substantially decrease the antimicrobial activity of film coatings.^17^

Carbonated hydroxyapatite (CHA) is an inorganic substance that promotes remineralization of hard tissues and exhibits some degree of antimicrobial activity.^18^ CHA has been used to incorporate nanoparticles and may contribute to physicochemical stability aiming to enhance antimicrobial activity. They immobilize the nanoparticles in their pores reducing the aggregates formation.^19^ In addition, CHA shares both chemical and mechanical similarities with teeth and bone tissues, presenting biocompatibility, osteoconductive and good response to biological pH values. CHA has shown promising results in studies involving stem cell differentiation, bone tissue engineering and dental applications.^20–22^ Furthermore, CHA may benefit from surface functionalization by adding antimicrobial substances like AgNPs to increase their osteoconductive effectiveness while promoting the biofilm control. Langmuir–Blodgett (LB) film technology is an advanced method used to create an ultrathin biomimetic lipid membrane at the molecular level.^23^ Literature indicates that phospholipid-based coatings are promising for the development of bioactive coatings the metal surfaces.^24^ The use AgNPs associated with CHA in dental products is quite limited and mainly related to dental composites.^25^ There is still a lack in the literature on studies evaluating the effect of functionalization of titanium surfaces with phospholipidic films containing AgNPs associated with CHA and their effect on the odontoblast proliferation, bacterial biofilm formation and antimicrobial activity.

In this investigation, we have created innovative bifunctional phospholipidic film coatings on titanium surfaces aiming to reduce the microbial attachment and proliferation without compromising the osteoblast affinity. This study is intended to show a significant reduction of the oral biofilm formed on titanium surfaces. We hypothesized that AgNPs-conjugated CHA (AgNPs/CHA) coatings would significantly reduce the biofilm formation on the titanium while maintaining their osteoconductive. This was achieved by incorporating CHA coating, containing or not AgNPs, on a grade 2 titanium used for dental implants.

## 2 Experimental Section

### 2.1 Materials and Study Design

This in situ study was conducted using titanium discs. Pure titanium discs (10 mm diameter and 1.0 mm thickness, grade 2) were purchased from Realum Ltd. (São Paulo, Brazil) and mechanically polished to grit levels of 1200 using a sequence of abrasive papers with increasing grit size (#320, #400, #600, and #1200) under water irrigation until a smooth surface ranging from 0.2–0.3 μm was obtained. After, the discs were sonicated during 10 in the following solutions: sodium dodecyl sulfate detergent in deionized water, acetone and absolute ethanol. Then, discs were dried at 40 °C for 1 h and stored in a vacuum desiccator. All the discs followed this protocol and served as the base substrate for the deposition process of phospholipid-based coatings in the experimental groups. The polished titanium discs were randomly assigned (using Microsoft Excel) into 4 different groups (n = 9) according to the surface treatment, as follows: Polished - no surface treatment, which is denoted as control group; SAE - sandblasted and double acid-etched, simulating the widely used surface modification for improving osseointegration; PCHA - phospholipidic film with CHA; and AgNPs/PCHA - phospholipidic film with AgNPs-conjugated CHA. The sample size estimation was based on the microbial count effect size of raw data from similar previous studies. ANOVA-like effect size estimated by Omega squared (ω^2^) resulted in 0.59, considering 4 independent groups in a one-way factorial design. The minimal significance (α) and statistical power (1 – β) were set at 0.05 and 0.80 respectively.

The microbiological tests were carried out by means of an in situ-controlled split-mouth design. For this, nine healthy participants were recruited from the student population at the Ribeirão Preto School of Dentistry, University of São Paulo, Brazil (mean age: 25 ± 3 years). Inclusion criteria included the presence of all teeth in the maxilla and no signs or symptoms of oral pathologies. Exclusion criteria included individuals under 18 years of age, pregnancy or breastfeeding, smoking, systemic conditions, active carious lesions, periodontal disease, and the use of antibiotics or medications affecting periodontal status within the past three months. All participants received both written and verbal information regarding the study objectives and procedures. The study protocol was reviewed and approved by the Research Ethics Committee of the School of Dentistry of Ribeirão Preto (CAAE: 66256922.6.0000.5419).

### 2.2 Sample Preparation

The polished group was designated as the control group and no further surface treatment was applied. In the other 3 groups, the specimens’ surfaces were treated as follows.

#### Sandblasting and Double Acid-Etching (SAE)

sandblasting was performed with 100 μm aluminum oxide particles using a laboratory sandblaster with 90 °C nozzle angle operated at 0.45 MPa pressure, 30 mm distance and 30 s exposures. The treated surfaces were washed with deionized water for 30 s and ultrasonically cleaned with a mixture of 100 mmol/L KH_2_PO_4_/NaOH (pH 7.5) and Span 20 (4 × 10^–5^ mol/L) at 65 °C for 5 min. After air-drying, double acid etching was performed by immersing discs in 18% HCl at 60 °C for 15 min followed by washing with deionized water and immersion in 49% H_2_SO_4_ at 60 °C for 15 min. Next, the discs were sonicated with deionized water for 15 min and dried at room temperature.^26^

#### Phospholipidic Film with CHA (PCHA)

Langmuir monolayer deposition was carried out by recording surface pressure (π)–area (A) isotherms at 25.0 ± 0.5 °C using a 216 cm^2^ Langmuir trough. A 30 μL aliquot of a 1.0 mmol L^–1^ dihexadecyl phosphate (DHP) solution, prepared in HPLC-grade chloroform/methanol (3:1, v/v), was carefully spread onto the subphase. The subphase consisted of 140 mL of an aqueous CaCl_2_ solution (1 mmol L^–1^). Calcium ions were included in the subphase solutions to promote adhesion between the phospholipid layers of DHP on the LB films and to act as a primary source of calcium ions for subsequent mineralization. The monolayers were deposited on the discs by the LB technique.^27^ Prior to coating, discs were cleaned under ultrasound in 100 mmol/L KH_2_PO_4_/NaOH (pH 7.5) and Span 20 (4 × 10^–5^ mol/L) at 65 °C for 5 min, followed by rinsing with deionized water. First, discs were immersed in the subphase containing 1 mmol/L CaCl_2_ solution followed by spreading the phospholipid solution. The DHP monolayers were deposited on the discs in 2 immersion cycles (withdrawal: 1 layer and immersion: 2 layers) until achieved LB films with 6 DHP monolayers. The immersion/withdrawal rate was 0.038 mm/s; the surface pressure (π) was kept at 30 mN/m. Hydrophilic films were formed on the discs because of the phosphate group of the phospholipid exposed in the outermost layer.

Mineral deposition onto the LB film was carried out in two sequential steps. First, the coated discs were subjected to a calcium/phosphate buffer cycle, which involved immersion in a 1.0 mmol/L^–1^ Ca^2+^ aqueous solution for 12 h, followed by immersion in a phosphate buffer solution (KH_2_PO_4_/NaOH, pH 7.5) for another 12 h. This cycle was repeated four times to ensure efficient binding of Ca^2+^ ions to the negatively charged phosphate groups of the phospholipid films and to promote local supersaturation at the interface. In the second step, the discs were immersed for 36 h in simulated body fluid (SBF) solution, a well-established method for assessing surface bioactivity, as it mimics the *in situ* formation of apatite layers on various material surfaces. All mineralization procedures were performed at 37 °C to simulate physiological conditions.^27^

#### Phospholipidic Film with AgNPs-Conjugated CHA (AgNPs/PCHA)

AgNPs were synthesized by reducing AgNO_3_ with NaBH_4_. Briefly, 10 mL of 1 mmol/L AgNO_3_ was added to 40 mL of 2 mmol/L NaBH_4_ under vigorous stirring. The formation of a yellow colloidal suspension indicated nanoparticle synthesis, which UV–Vis spectroscopy confirmed. Particle size and zeta potential were analyzed by Dynamic light scattering (DLS). Subsequently, the DHP monolayers and mineral deposition were prepared as previously described, with the addition of 1 mL of AgNPs solution and 139 mL of CaCl_2_ solution to the subphase (the theoretical calculation of silver concentration in the subphase is provided in the Supporting Information).

### 2.3 Titanium Surfaces Characterization

Surface morphology was examined using scanning electron microscopy (SEM) (JEOL JSM-6610; n = 2). Specimens were mounted on metallic stubs and sputter-coated with a thin gold layer under a pressure of 1 × 10^–5^ Torr. Cross-sectional SEM analysis was additionally conducted to determine the final thickness of the deposited hybrid films. For this purpose, samples were cryofractured in liquid nitrogen and subsequently sectioned using a lathe. The images were acquired under high vacuum conditions with an accelerating voltage of 15 kV. Elemental composition was determined by energy-dispersive spectroscopy (EDS) operated in conjunction with SEM and used to calculate the percentage of chemical elements within a penetration depth of approximately 2 to 5 μm.

The molecular structure and chemical groups formed on the titanium surfaces were identified by Fourier-transform infrared spectroscopy (FTIR) using an ATR accessory (Shimadzu IRPrestige-21) in the range of 400–4000 cm^–1^ (n = 2). For the nanoscale topographical analysis, atomic force microscopy (AFM) was performed using a Park Systems NX10 instrument (n= 2), operated in noncontact (tapping) mode under ambient conditions. X-ray photoelectron spectroscopy (XPS; Thermo Scientific, K-Alpha) was used to evaluate the chemical state of the oxide layer and determine the elemental composition within the outermost 1–10 nm of the surface. Spectra were acquired using monochromatic Al Kα radiation (1486.6 eV), with charge compensation and pass energy of 50 eV for high-resolution scans. Additionally, Inductively Coupled Plasma Optical Emission Spectroscopy (ICP-OES) was employed to detect and quantify the concentration of silver in titanium coatings and to investigate the release profile of silver ions over time. Briefly, coated samples were immersed in artificial saliva for two time points: 24 and 48 h. Following immersion, the samples were diluted at a ratio of 1:10 (w/w) with ultrapure water and then acidified with 1% (v/v) nitric acid (HNO_3_) to facilitate the extraction of silver ions. The concentration of silver was quantified using a calibration curve constructed with control artificial saliva, which was included to mitigate matrix effects and enhance the accuracy and reliability of the analytical results. The Limit of Detection (LoD) was determined to be 0.03 mg/kg.

The surface free energy (SFE) was determined by measuring the contact angles (θ) of three different liquids (diiodomethane, water, and formamide) on the experimental surfaces (n = 9) using a goniometer (DataPhysics OCA20). Linear parameters of surface roughness (n = 9) were quantified using laser scanning confocal microscopy (LSCM, VK-X200, Keyence) with a 50× objective lens and 1× digital zoom. Three measurements were taken along three arbitrary radial lines with a cutoff of 80 μm and an evaluation length of 2.57 mm. The mean value of the three measurements was considered as roughness values of the respective sample.

The mechanical stability of the coatings was evaluated through adhesion testing of the LB films, following the procedures specified in IRAM 2454 (NM 60454–2:2006) and SABS standards. An insulating adhesive tape was applied and removed under controlled conditions to assess the adhesion performance. A 10 mm-wide adhesive tape with high affinity for polar substances was applied to titanium discs (n = 5) previously coated with the films. The tape was pressed using a 6 kg roller and left in contact for 20 min. It was subsequently peeled off at a 180° angle using a universal testing machine (BioPDI) equipped with a 50 kg load cell, operating at a constant extension rate of 300 mm/min. The results were reported in gram-force units (gf; 1 gf = 0.01 N).

### 2.4 Cell Proliferation Assay

The MTT colorimetric assay [3-(4,5-dimethylthiazol-2-yl)-2,5-diphenyl tetrazolium bromide] was used to assess the survival and proliferation of osteoblasts cells on the titanium discs coated with LB films containing either CHA or AgNPs-conjugated CHA. To evaluate the cytotoxicity of the surface treatments (n = 5), 2 × 10^4^ MC3T3-E1 osteoblast-like cells suspended in 1 mL of culture medium were seeded in each well of a 24-well plate and incubated for 5 days. After the incubation period, 1 mL of MTT solution (1.0 mg/mL) was added to each well and the plates were incubated for 4 h at 37 °C. The resulting formazan crystals were dissolved in 2-propanol and agitated to ensure complete solubilization. The absorbance was measured at 570 nm using a microplate reader and the number of viable cells was estimated based on the amount of dissolved formazan product.

### 2.5 Contamination Test

Acetate plates (1 mm thickness) were heated and molded over the upper gypsum cast obtained from the participants using a vacuum-forming machine (Plastvac P7, Bio-Art) and used to expose titanium discs in the oral cavity. Four discs (one from each experimental group) were randomly fixed onto the premolar and molar regions of the intraoral device using colorless self-curing acrylic resin. Volunteers were instructed to wear the devices continuously for 48 h, removing only for eating and oral hygiene. While not in use (e.g., during meals), the devices were immersed in 250 mL of 0.9% saline solution to prevent dehydration while maintaining microbial viability. During the experimental period, they were advised to avoid the use of antimicrobial agents and alcoholic beverages. After meals and oral hygiene, participants were instructed to reinsert the device without brushing or cleaning the discs surfaces.

### 2.6 Morphological Analysis of Microorganisms Adhered to the Discs

The morphology of microbial species colonizing the formed biofilm on the experimental surfaces (n = 2) were evaluated by scanning electron microscopy (SEM). Contaminated discs were removed from the intraoral devices, washed with phosphate-buffered saline (PBS) solution and fixed in 2.5% glutaraldehyde solution diluted in PBS for 2 h at room temperature. After fixation, the samples were washed with PBS and dehydrated through a graded ethanol series (50%, 60%, 70%, 80%, 90%, 95%, and 100% v/v; 10 min in each step). The discs were then mounted on metallic stubs and sputter-coated with a thin layer of gold under a pressure of 1 × 10^–5^ Torr and a voltage of 25 kV. Analyses were carried out in three randomly selected regions of each sample group.

### 2.7 Live/Dead Immunofluorescence Analysis

The structural organization of biofilm formation on the titanium discs was evaluated through substrate coverage and biovolume analysis using the Live/Dead immunofluorescence assay. Following intraoral exposure, the discs were immediately removed from the devices and washed with PBS solution to eliminate nonadherent cells. Biofilms were stained with SYTO 9 (FilmTracer, Invitrogen) to identify viable cells and with propidium iodide to label nonviable cells. nm for propidium iodide. To quantify the surface area covered by live and dead cells, the stained discs were examined using a fluorescence microscope (Axio Observer A1, Carl Zeiss, Germany) equipped with FITC and RHOD filters to detect green- and red-stained cells, respectively. Ten randomly selected fields were analyzed per sample (n= 2). Images were acquired at 63× magnification and processed individually using Zen Lite 2.3 software (Carl Zeiss). Quantification of the covered surface area was performed using AxioVision software (release 4.2, Carl Zeiss). A total of 160 randomly selected images of the biofilm-covered surfaces were analyzed. The red and green-stained regions, representing nonviable and viable bacterial cells, respectively, were converted into percentage values using the following formula: marked area/total image area × 100. To calculate the biovolume, stained samples were observed using a confocal laser scanning microscope (Leica TCS SP8). Two detection channels were used: channel 1 for red-stained (dead) cells and channel 2 for green-stained (viable) cells. Five randomly selected fields were analyzed per sample (n = 6). Images were acquired at 63× magnification and the biovolume quantification was performed using the Biofilm Architecture Inference Tool (BAIT) through the BEM thresholding segmentation method.^28^

### 2.8 Checkerboard DNA–DNA Hybridization Analysis

Twenty-five bacterial species, including both pathogenic and nonpathogenic microorganisms, were selected as the target species (*Bacteroides fragilis, Enterococcus faecalis, Pseudomonas aeruginosa, Campylobacter rectus, Streptococcus gallolyticus, Porphyromonas endodontalis, Staphylococcus aureus, Treponema denticola, Streptococcus mutans, Campylobacter gracilis, Tannerella forsythia, Lactobacillus casei, Streptococcus mitis, Aggregatibacter actinomycetemcomitans, Capnocytophaga gingivalis, Peptostreptococcus anaerobius, Streptococcus sanguinis, Streptococcus salivarius, Mycoplasma orale, Lactobacillus acidophilus, Pseudomonas putida, Klebsiella pneumoniae, Prevotella intermedia, Porphyromonas gingivalis,* and *Prevotella melaninogenica*. After contamination test, the entire biofilm formed on the surfaces of titanium discs from the four groups (n= 9) was individually collected using a regular microbrush and transferred into microtubes containing 150 μL of TE buffer (10 mM Tris-HCl, 1 mM EDTA, pH 7.6) followed by addition of 150 μL NaOH 0.5 M. The microtubes containing the samples were vortexed for 2 min to disaggregate the contents. The samples were thermally denatured by heating at 98 °C for 5 min. Immediately after heating, the microtubes were transferred to ice and added 800 μL of 5 M ammonium acetate. The samples were processed according to do Nascimento et al. (2010).^29^ Species identification and quantification were performed using CLIQS – Core Laboratory Image Quantification software (TotalLab).

### 2.9 Statistical Analysis

The assumptions of normality and homoscedasticity of residuals were assessed for quantitative variables using histograms and scatterplots and confirmed by significance tests. When these assumptions were met, one-way analysis of variance (ANOVA) was applied followed by Tukey’s post hoc test for multiple comparisons. In case of violations, Kruskal–Wallis test followed by Dunn’s post hoc test corrected by Bonferroni was applied. Linear regression was applied to estimate the magnitude of differences between groups in the wettability assay. For the microbiological outcomes, statistical models accounting for hierarchical and dependent data were used. Initially, outcomes with paired or repeated-measures structure were evaluated using the nonparametric Friedman test followed by Nemenyi’s post hoc test for multiple comparisons. Furthermore, to estimate the magnitude of group effect, generalized linear mixed models (GLMMs) were fitted using either the Tweedie distribution with a log link or negative binomial distribution, depending on the nature and variability of the data. These models appropriately accounted for intrasubject correlation and the high dispersion observed in the data. All analyses were performed at a 5% significance level (*p* < 0.05) using R software (version 4.2.3) with the *rstatix*, *lme4*, *glmmTMB*, and other relevant packages.

## 3 Results and Discussion

### 3.1 Characterization of AgNPs

Figure 1A presents the UV–vis spectrum of the colloidal silver particles. The formation of AgNPs was confirmed by the presence of the absorption plasmon peak around 390 nm. This peak is characteristic of the collective oscillation of conducting electrons on the nanoparticle surfaces in the presence of a light wave field. The particle size distribution presented in terms of number indicated a monodisperse system with an average AgNPs diameter of 3.887 nm (Figure 1B). The zeta potential of the colloidal AgNPs suspension was −19.9 mV (Figure 1C). The negative potential of AgNPs prepared by the chemical reduction of silver nitrate with sodium borohydride is due to the adsorption of BH_4_^–^ radicals on the nanoparticle surfaces, which electrostatically stabilizes the NPAg. This magnitude of zeta potential indicates moderate electrostatic stabilization; aggregation remains likely under some conditions.^30^

**Figure 1:**
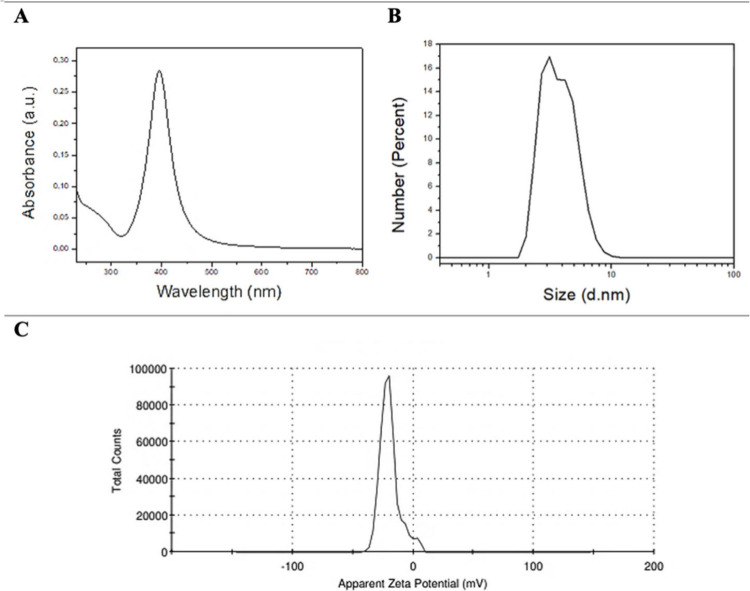
(A) UV–vis absorption spectrum indicating the colloidal monodispersed of nanoparticles. (B) Particle size distribution. (C) Zeta potential distribution.

### 3.2 Monolayers Characterization

The isotherms shown in Figure 2A illustrate the behavior of DHP molecules at the air/water interface under different surface pressures. Initially, the dispersion of DHP phospholipids from the chloroform solvent resulted in a reduction in the interfacial tension allowing the spontaneous spreading of the molecules. After solvent evaporation, the DHP molecules remained anchored to the aqueous subphase by their polar head groups due to electrostatic interactions. Simultaneously, repulsive forces between water molecules and the hydrophobic tails of the phospholipids promote the alignment of hydrocarbon chains through hydrophobic interactions. These forces are sufficiently weak to avoid the lateral aggregation and permit the spontaneous spreading of the DHP. The presence of dissolved electrolytes in the subphase may have influenced the interfacial organization of phospholipids, as represented by the blue curve in Figure 2A. The addition of Ca^2+^ ions and AgNPs to the interface reduced the molecular area and resulted in a more compact monolayer (red curve in Figure 2A), indicating interactions between the phospholipids and the nanoparticles at the interface between the polar headgroups and the aqueous phase, which minimizes lateral repulsion. According to the range of *C*_*S*_^‑1^ values in Figure 2B, the monolayers were presented in the liquid-condensed phase, considered optimal for monolayer transfer onto titanium surfaces.^31^

**Figure 2:**
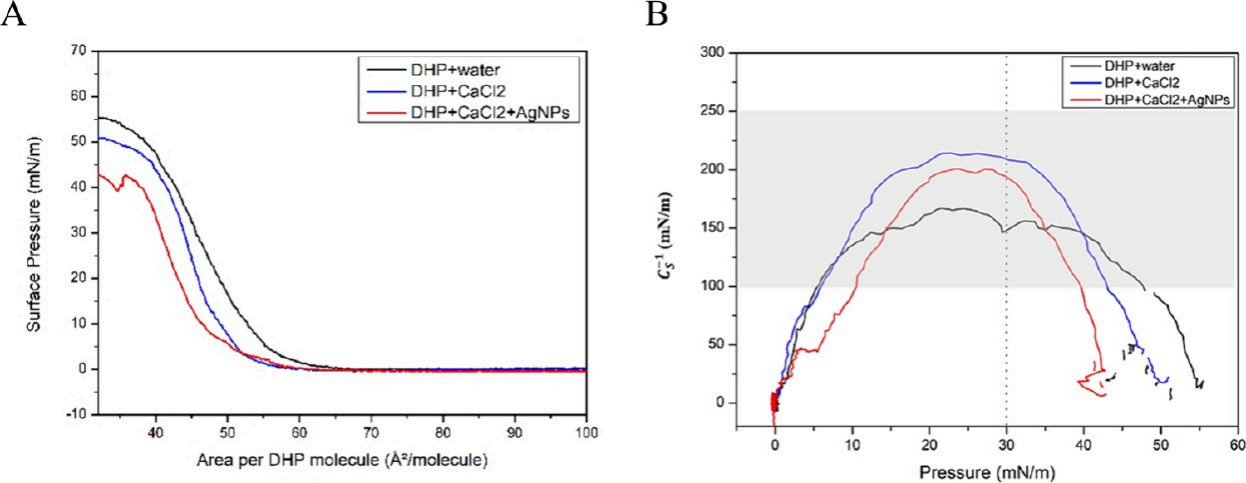
(A) Surface pressure isotherm as a function of molecular area obtained for DHP on ultrapure water, CaCl_2_ solution and CaCl_2_ solution containing AgNPs. (B) Compression modulus calculated for the monolayers formed on ultrapure water, aqueous CaCl_2_ solution, and CaCl_2_ solution containing AgNPs. The dashed line indicates the deposition pressure, and the highlighted region corresponds to the liquid-condensed phase range.

Figure 3 illustrates the morphological analysis of monolayer formation on different subphases performed by Brewster angle microscopy (BAM). The spreading of DHP was notably greater without Ca^2+^ or AgNPs. The presence of calcium ions and silver nanoparticles have reduced the electrostatic repulsion between polar headgroups and promoted the phospholipid aggregation. At surface pressures of 10, 20, and 30 mN/m, the phospholipid molecules have exhibited dense packing as evidenced by the increasingly distinct gray contrast against the black background, suggesting that the DHP molecules are well-organized with their hydrophobic tails oriented toward the air interface.^32^ Overall, BAM images have showed an increased number of bright spots in the monolayer formed over the AgNPs-containing subphase, reflecting increased film thickness.^33^ The additional monolayer compaction in the presence of AgNPs associated with the increased thickness observed by BAM suggests electrostatic interactions between the polar headgroups of the phospholipids and the surface of the silver nanoparticles.

**Figure 3:**
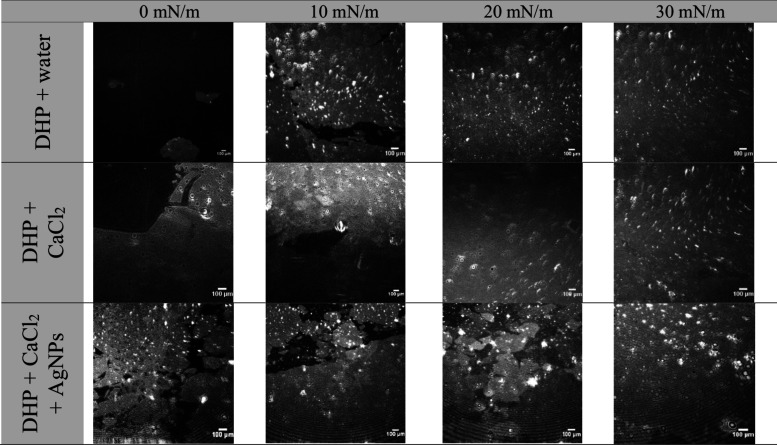
Morphological analysis of monolayer formation on different subphases performed by Brewster angle microscopy (BAM) equipped with a 10× objective.

### 3.3 Surface Characterization: FTIR, XPS, EDS, and ICP-OES Analyses

Figure 4 presents the FTIR spectra of functional groups on the surface of the discs. The narrow band at ∼1058 cm^–1^ corresponds to the asymmetric stretching (ν_3_) of the PO_4_^3–^ group. The weak bands between 1400–1526 cm^–1^ are associated with the vibrational modes (ν_3_) of CO_3_^2–^. The bands around 1400 cm^–1^ indicate B-type carbonated hydroxyapatite, while the band at ∼1526 cm^–1^ suggests A-type carbonate substitution. Additional broad and narrow absorptions at ∼3300 cm^–1^ and ∼1633 cm^–1^ are associated with hydroxyl groups and/or adsorbed water. These results support the formation of A-type carbonated hydroxyapatite in the PCHA discs and a mixed A/B-type carbonate substitution in the AgNPs/PCHA discs.^31^ The band at ∼1058 cm^–1^, related to PO4^3–^ v_3_ was broadened and slightly displaced to lower wavenumber in the presence of AgNPs. Similar alterations in phosphate bands have been reported for hydroxyapatite/silver composites and for phosphate-based materials with ionic substitutions, indicating that the incorporation of Ag can modify the local chemical environment and the vibrational behavior of phosphate groups. These effects are commonly interpreted as resulting from changes in the hydrogen-bond network, vibrational couplings between adjacent groups, or partial coordination of phosphate oxygens with cations (e.g., Ca^2+^) and/or adsorbed silver species.^56^

**Figure 4:**
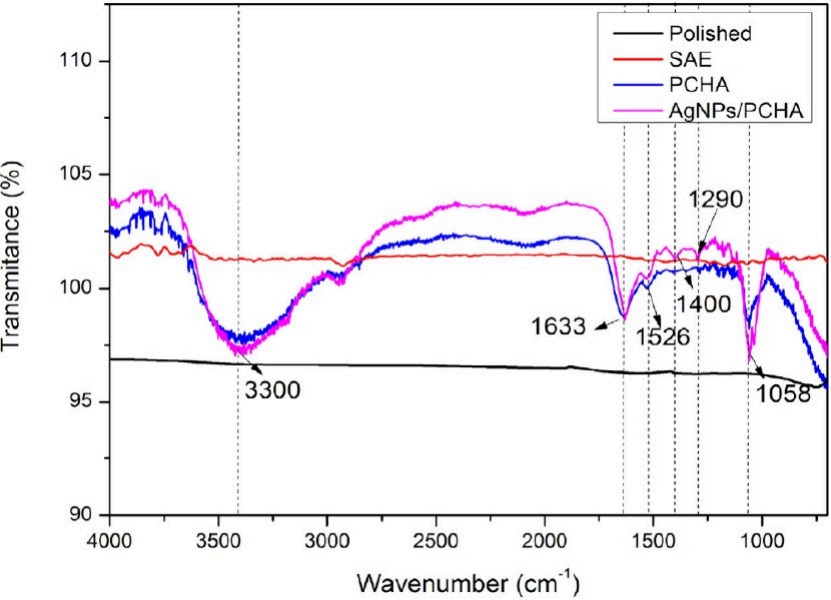
FTIR spectra of the surfaces of titanium discs after different surface treatments.

Table 1 displays the elemental distribution within the uppermost layers of the discs (1–10 nm), as obtained from the XPS mapping spectra. Carbon was the most abundant element detected in the outermost layers of all tested surfaces, followed by oxygen. In the Polished and SAE discs, the carbonaceous material originates from a thin layer formed upon air exposure, commonly referred to adventitious carbon, which is typically composed of hydrocarbons and lower amounts of oxygen-containing functional groups such as single or double bonds.^34^ Nitrogen, calcium, and silicon were also detected and may be attributed to the atmospheric contamination, such as vapor or ambient aerosol, often present as carbonates and silicon oxides.

**Table 1:** Chemical Composition (%) of Experimental Groups Characterized by XPS Analysis^a^

	Polished	SAE	PCHA	AgNPs/PCHA
**C**	84.61	74.28	68.91	67.34
**O**	11.84	17.57	21.66	21.42
**Ca**	2.3	0.91	2.16	2.32
**N**	0	0.58	1	1.19
**Si**	1.12	5.03	1.08	1.06
**Na**	0	0	0.41	0.52
**Cl**	0	0	0.05	0.94
**P**	0	0	2.18	0.67
**B**	0	0	0	1.87
**Ti**	0.12	1.63	2.55	2.67

a SAE: sandblasted and double acid-etched; PCHA: phospholipidic film with CHA; AgNPs/PCHA: phospholipidic film with AgNPs-conjugated CHA.

On the surfaces of the PCHA and AgNPs/PCHA discs, the presence of carbon (C) and oxygen (O) suggests the incorporation of phospholipid hydrocarbon chains and carbonate species. The detection of chlorine (Cl) and sodium (Na) is commonly associated with biomimetic carbonated hydroxyapatite formation, either through ionic substitution within the apatite lattice or as surface-associated species originating from SBF-based synthesis. The ionic substitutions occur spontaneously during nucleation and crystallization from a defect-rich amorphous phase, being favored by the high ionic strength and biomimetic composition of the medium. Na^+^ occupy calcium sites, promoting structural disorder, a reduction in lattice parameters, and increased similarity to biological apatite. Cl^–^ is associated with the biomimetic environment of the coating, likely absorbed or weakly bound to the hydroxyl channel region of hydroxyapatite and/or to the hydrated layer of the phospholipid film.^35^

The presence of boron (B) in the AgNPs/PCHA coatings is consistent with the zeta potential data and suggests the adsorption of borohydride ions on the surface of the nanoparticles. Based on the synthesis stoichiometry, the estimated upper-bound concentration of boron in the AgNP colloid is approximately 15 mg·L^–1^, corresponding to approximately 15 μg of boron in the 1.0 mL aliquot added to the LB subphase. Upon deposition, this results in a theoretical dilution of ≈0.108 mg·L^–1^ in the final subphase (Supplementary S2). These boron concentrations are several orders of magnitude lower than those typically associated with antimicrobial or cytotoxic effects, where minimum inhibitory concentrations (MIC) are reported to range between 0.3 and 10 mg·mL^–1^. Consequently, it is reasonable to conclude that any potential contribution from residual borohydride is negligible and unlikely to influence the observed biological or material properties.

The high-resolution C 1s spectra (Figure 5) of the PCHA and AgNPs/PCHA discs revealed a peak at 284.8 eV attributed to C–C and C–H bonds, indicating the presence of phospholipid hydrocarbon chains within the hybrid coatings. The peak at ∼288 eV (O–C=O) was associated with the presence of carbonate (CO_3_^2–^) species from hydroxyapatite coatings.^36^ The C–O component observed at around 286 eV further supports the presence of the DHP phospholipid, likely arising from interactions involving phosphate-containing groups.

**Figure 5:**
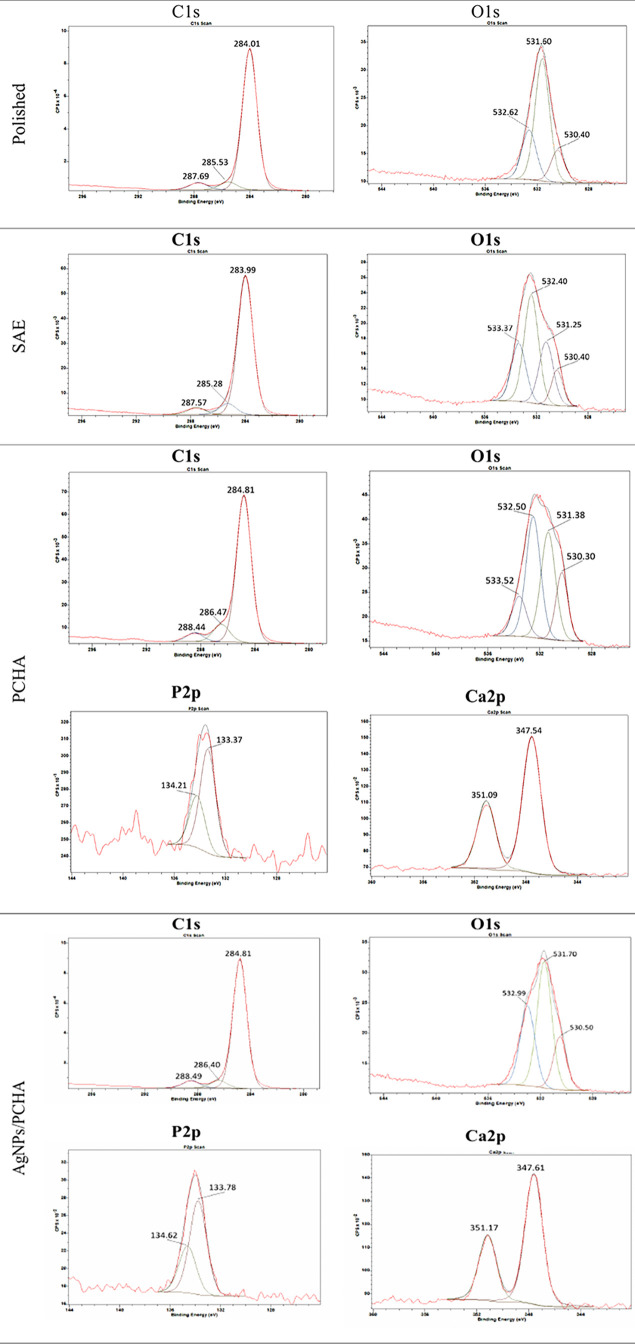
Peak fitting of high-resolution XPS spectra for the C 1s, O 1s, P 2p and Ca 2p orbitals.

The high-resolution O 1s spectra of the Polished discs have shown a peak signal at ∼530 eV, indicating the presence of a passive film composed of TiO_2_ on the titanium surface (Figure 5). A less intense peak at ∼532 eV corresponds to oxygen bonded to adventitious carbon. XPS analysis has revealed four distinct oxygen chemical states on the SAE discs. Two types of metal oxides were identified: TiO_2_ (∼530 eV) and Ti­(OH)_*x*_ (∼531 eV), the latter resulting from the dual acid etching process. This treatment has induced extensive surface hydroxylation and has increased the microscale roughness. Additional peaks recorded at ∼532 eV and ∼533 eV were attributed to oxygen species associated with atmospheric contaminants.

On the surfaces of the PCHA and AgNPs/PCHA discs, a hybrid film was formed over the TiO_2_ as evidenced by the peak at ∼530 eV. On the PCHA surface, a high oxygen content (53.64%) was primarily associated with the peak at ∼ 531 eV, corresponding to oxygen–phosphorus bonds in the PO_4_^3–^ moiety. The peak at ∼532 eV was attributed to carbonate groups (CO_3_^2–^), representing 24.06% of the total oxygen signal. The coatings applied to the AgNPs/PCHA discs resulted in a distinct O 1s spectral profile with oxygen signals distributed between phosphate (39.9% at ∼531 eV) and carbonate (39.48% at ∼532 eV) species. The increased carbonate content observed in the AgNPs/PCHA discs corroborates with the FTIR results and supports the formation of carbonated hydroxyapatite with mixed A- and B-type substitutions.^35^ High-resolution spectra of the Ag 3d orbital were analyzed to confirm the presence of silver nanoparticles on the outermost surface of the AgNPs/PCHA discs (Figure 6). Peaks were identified at ∼368 eV and ∼374 eV, corresponding to the Ag 3d_5_/_2_ and Ag 3d_3_/_2_ orbitals, respectively. When silver was present at very low concentrations, the number of photoemitted electrons decreased, resulting in a higher signal-to-noise ratio.

**Figure 6:**
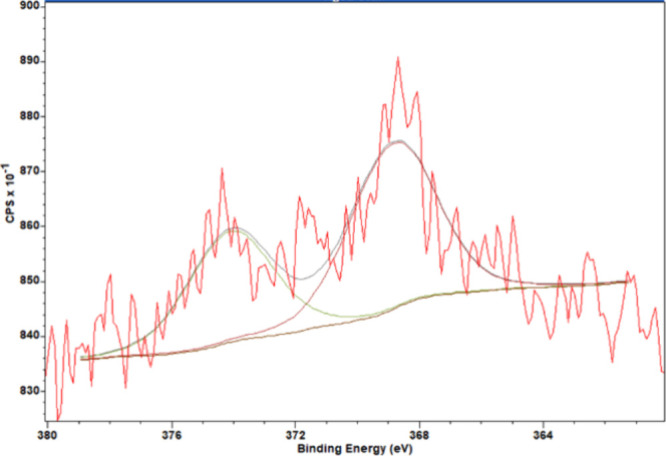
Peak fitting of the high-resolution XPS spectrum of the Ag 3d orbital.

The EDS analysis (Table 2) identified titanium as the dominant element in all groups, indicating that the chemical modifications were restricted to the surface layer and did not affect the underlying substrate. The polished discs (control) were predominantly composed of titanium, consistent with a minimally modified surface, along with oxygen associated with the naturally formed titanium oxide layer. In contrast, the SAE discs exhibited increased oxygen content and the presence of sulfur, suggesting modification and partial dissolution of the native oxide layer, as well as the formation of a new surface film with altered morphology. The PCHA discs exhibited a more complex elemental composition, characterized by elevated levels of oxygen and carbon, consistent with the presence of an organic matrix rich in phospholipids, together with detectable amounts of calcium and phosphorus. The presence of calcium and phosphorus confirms the formation of a carbonated hydroxyapatite layer on the surface, while the relatively higher calcium content suggests the development of a denser mineral coating.

**Table 2:** Surface Chemical Composition of Discs Determined by EDS (%)

	C	O	Si	S	Cl	Na	K	Ca	P	Ag	Ti
**Polished**	1.34	8.73	0.87	–	–	–	–	–	–	–	89.38
**SAE**	–	13.66	–	0.31	–	–	–	–	–	–	86.03
**PCHA**	4.12	13.6	–	–	1.59	2.16	0.1	0.24	0.17	–	77.95
**AgNPs/PCHA**	2.38	10.05	–	–	0.02	0.29	–	0.09	0.06	0.03	87.09

The AgNPs/PCHA discs exhibited a broadly similar elemental profile to the PCHA group; however, reduced carbon and oxygen contents were observed, in combination with the incorporation of silver into the coating. This finding indicates that the inclusion of silver nanoparticles may have influenced the phospholipid fraction and affected the density and organization of the mineralized layer (Ag:Ca ratio of approximately 0.33). The presence of sodium and chlorine was observed exclusively in the groups coated with biomimetic films containing carbonated hydroxyapatite. Sodium was identified by both XPS and EDS analyses, supporting its incorporation into the coating and suggesting a possible partial substitution of calcium within the apatite lattice, consistent with the behavior reported for biological apatites. In contrast, chlorine exhibited a distribution that depended on the coating architecture, suggesting a predominantly surface-associated presence and/or interactions with structural defects and the phospholipidic film.

ICP-OES analysis did not detect the presence of silver (Ag) in any of the samples, with all concentrations being below the Limit of Detection (LoD) of 0.03 mg/kg. This absence of detection suggests that silver nanoparticles were not present at measurable levels in the analyzed samples. The absence of silver lixiviation over time suggests that the silver nanoparticles remained stable and were not released from the coating into the surrounding environment. These findings are indicative of favorable biocompatibility, as the lack of silver release minimizes potential toxicity and adverse effects, supporting the coating’s suitability for applications where biocompatibility is a critical factor (Additional information on Supplementary S3).

### 3.4 Surface Morphology: SEM and ATM Analyses

Figure 7 illustrates the SEM images of titanium discs used in this investigation. The Polished discs exhibited a smooth surface with shallow polishing marks, whereas the SAE group displayed a hierarchical microstructure with pores of varying depths, resulting from sandblasting followed by acid etching. This acid treatment removed the native TiO_2_ layer and promoted the formation of a new oxide film characterized by increased roughness and an altered chemical composition, as previously confirmed by XPS. The experimental coatings (PCHA and AgNPs/PCHA) showed smoother and more uniform surfaces compared to Polished and SAE discs. In the PCHA discs, micrometric cubic crystals attributed to NaCl precipitated from the SBF solution were observed. Both experimental coatings formed continuous and homogeneous films that effectively minimized the initial surface irregularities.

**Figure 7:**
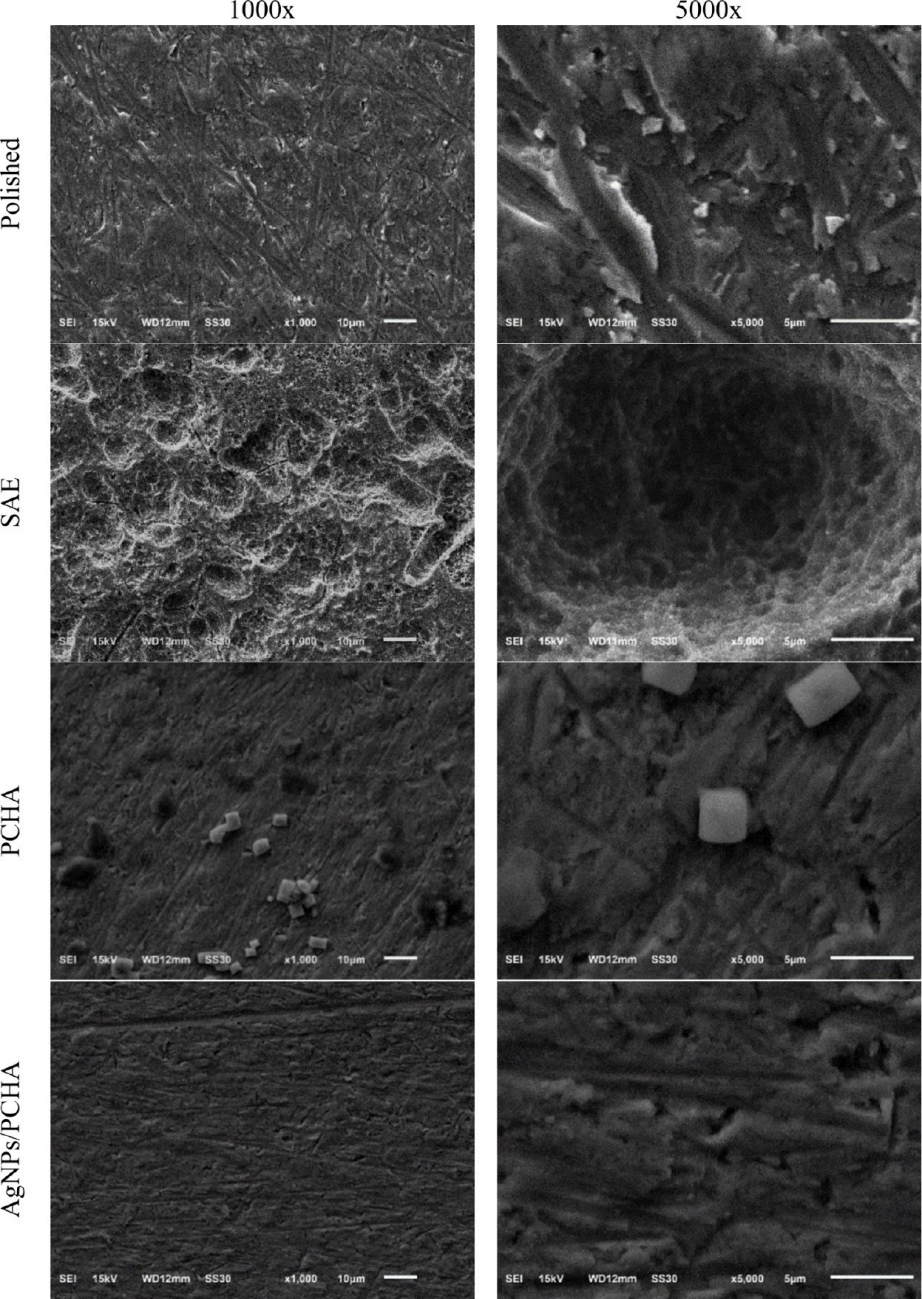
SEM imagens surface at different magnifications.

The thickness of the coatings determined from cross-sectional SEM images are illustrated in Figure 8. The measurements indicated a consistent coating thickness of approximately 1 μm, irrespective of the incorporation of silver nanoparticles. To assess the stability of the coatings under conditions mimicking oral environments, their morphology was examined after immersion in artificial saliva for 24 and 48 h, as shown in Figure 9. SEM analysis revealed no significant changes in the surface morphology of the coatings after these time periods, suggesting that the coatings remained stable and maintained their structural integrity under simulated oral conditions.

**Figure 8:**
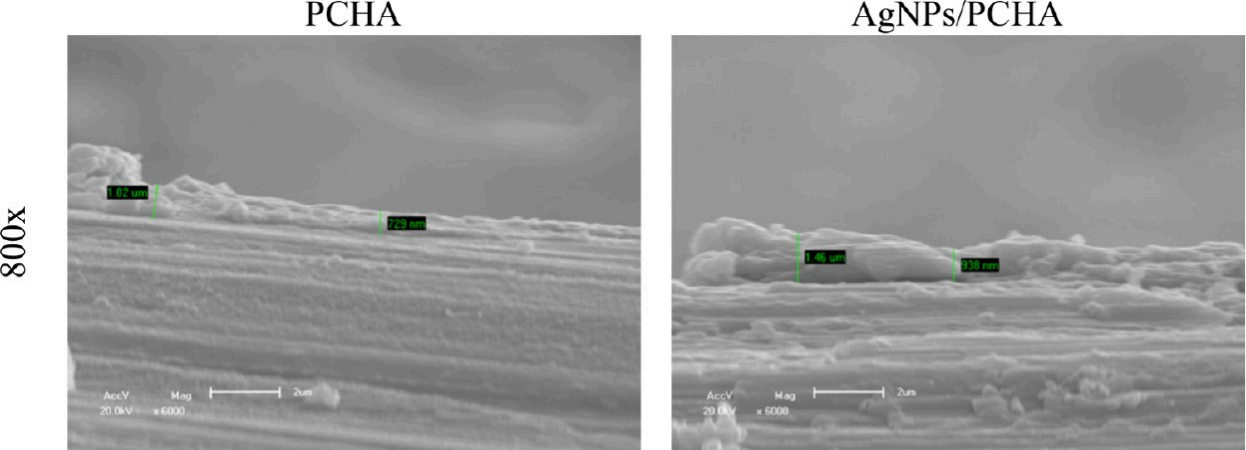
*Cross-sectional SEM images of the coatings.* The images show the thickness and uniformity of the coating (approximately 1 μm) with (AgNPs/PCHA) and without (PCHA) the incorporation of silver nanoparticles. The cross-sectional view illustrates the structural integrity of the coatings, with no noticeable defects or discontinuities observed (The scale bar represents 2 μm).

**Figure 9:**
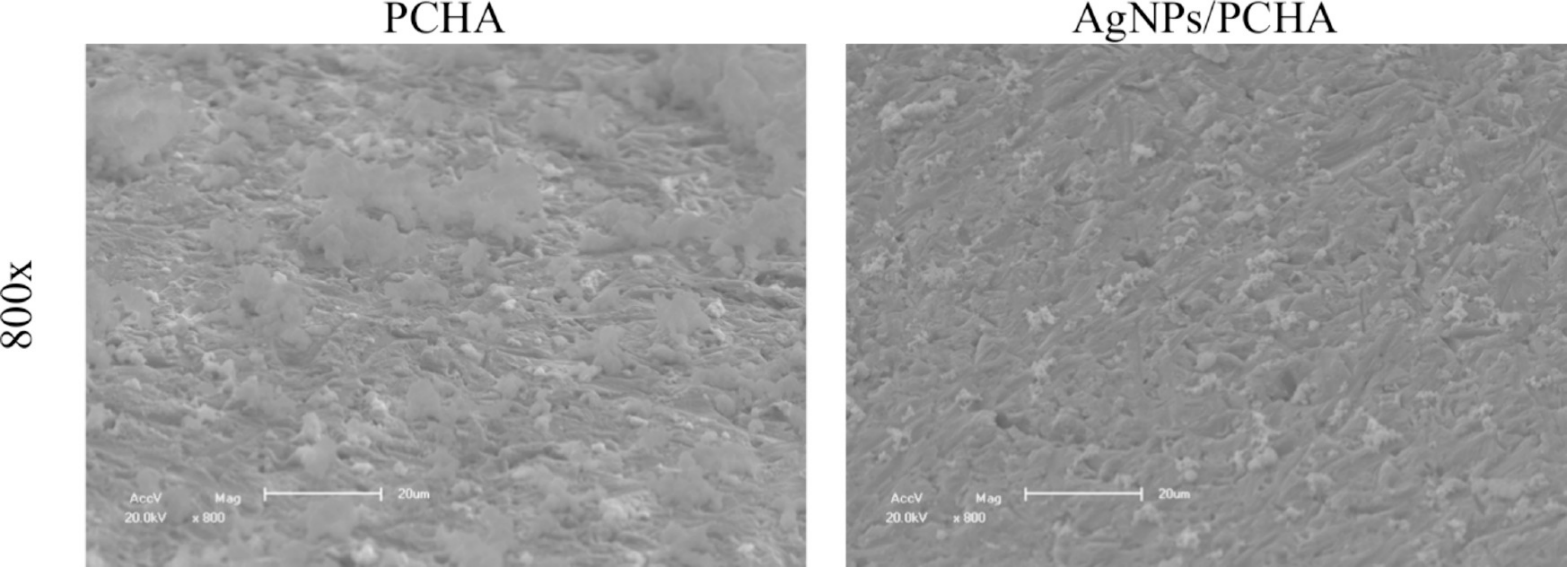
*Stability of the coatings after immersion in artificial saliva.* SEM images showing the surface morphology of the coatings with (AgNPs/PCHA) and without (PCHA) the incorporation of silver nanoparticles after immersion in artificial saliva for 48 h (the scale bar represents 20 μm).

Figure 10 illustrates the AFM images comparing experimental coatings against Polished control. PCHA and AgNPs/PCHA groups display submicrometric topographical features on the surfaces of the discs, characterized by densely packed particles. On the PCHA surface, dendritic particles were observed forming a branched network with a natural fractal-like pattern. In contrast, the AgNPs/PCHA surface exhibited a dense layer of spherical particles. The organic matrix plays a key role in modulating the crystallization process. The matrix alters molecular organization and charge distribution, favoring the formation of microdomains with different degrees of compactnes*s.* ^31,37^ The presence of the DHP phospholipid matrix plays a decisive role in the surface roughness of the samples. The successive deposition of monolayers promotes a gradual increase in coating thickness and density, which is also reflected in an initial rise in Ra values (Figure 9). This increase is associated with the formation of a more heterogeneous topography composed of partially ordered phospholipid domains, resulting in small height variations detected by AFM. The incorporation of AgNPs does not seem to produce noticeable changes in the surface topography. After the nucleation and growth of carbonated hydroxyapatite, a new modification in roughness parameters is observed. Ra tends to increase more markedly, reflecting the development of three-dimensional crystalline structures that emerge from the phospholipid matrix. This topographical evolution indicates that the matrix acts not only as a physical support but also as a mediator of the nucleation process, regulating the local supersaturation of calcium and phosphate ions as well as the orientation of the first crystalline nuclei. As the crystals grow, the surface becomes rougher and morphologically more complex.

**Figure 10:**
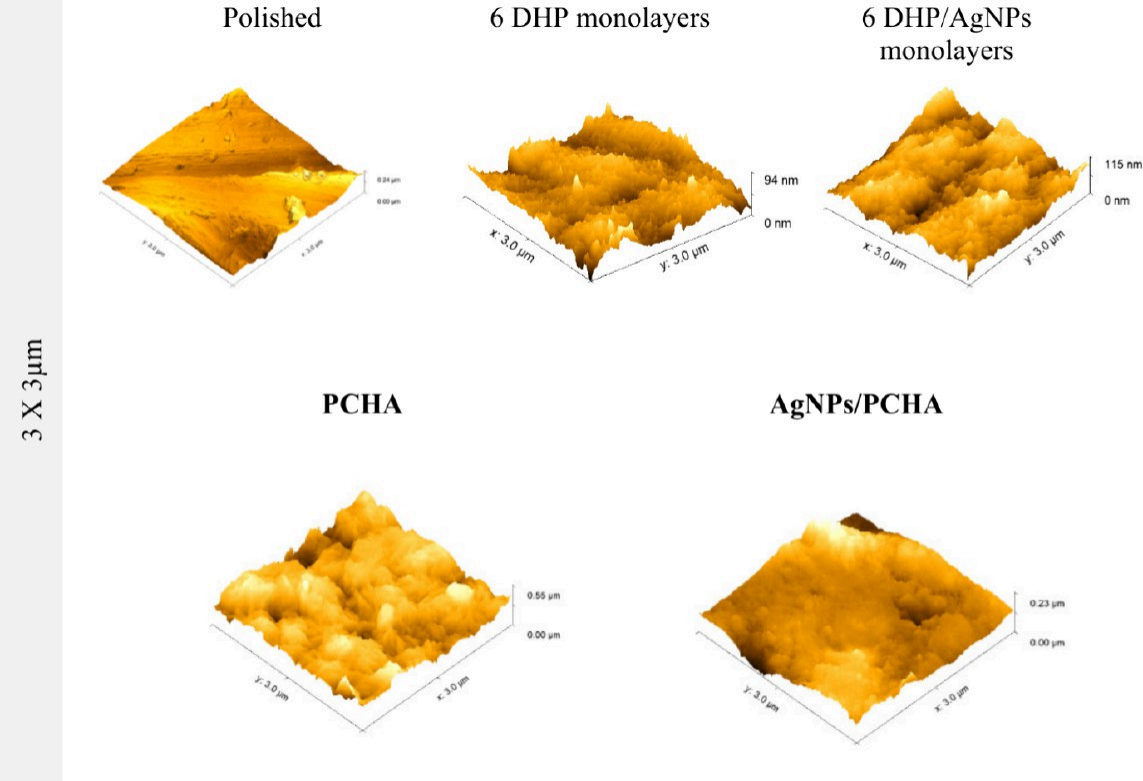
AFM images (3 × 3 μm^2^) and surface roughness parameters (Ra, in nanometers) obtained using Gwyddion version 2.65 with a cutoff of 0.3 μm. Quantitative analysis showed mean Ra values of 0.85 ± 0.15 nm for the polished surface, 1.07 ± 0.16 nm for 6 DHP monolayers, 0.92 ± 0.04 nm for 6 DHP/AgNPs monolayers, 2.83 ± 0.04 nm for PCHA, and 1.58 ± 0.17 nm for AgNPs/PCHA.

Therefore, the observed variations in Ra reflect different stages of structural organization: initially dominated by topographical fluctuations of the phospholipid matrix and later by the contribution of the crystalline phases formed upon it. This transition indicates that surface roughness is a sensitive parameter for tracking the evolution of physicochemical interactions occurring from monolayer assembly to the final hydroxyapatite growth.^38^

### 3.5 Surface Roughness

Polished and experimental coatings (PCHA and AgNPs/PCHA) discs have shown lower Ra and Rq values (Table 3), consistent with smoother surfaces as also observed in the SEM images. In contrast, the SAE discs showed significantly higher roughness due to surface modification by sandblasting followed by dual acid etching, which resulted in an irregular oxide layer with pronounced peaks (Rp) and valleys (Rv), as confirmed by 3D surface reconstructions (Figure 11). Despite the presence of a hybrid coating composed of a phospholipid matrix and carbonated hydroxyapatite, PCHA and AgNPs/PCHA have not shown significant changes in microscale roughness. However, AFM images revealed nanometric topographical changes in both experimental coatings. The slightly reduced Rv values found to PCHA and AgNPs/PCHA discs suggest that coatings partially smoothed polishing-induced surface irregularities. All the 4 evaluated groups presented Rku values around 2.5, indicating relatively uniform height distributions. These findings suggest that both PCHA and AgNPs/PCHA have produced uniform coatings without increasing microscale surface roughness.

**Figure 11:**
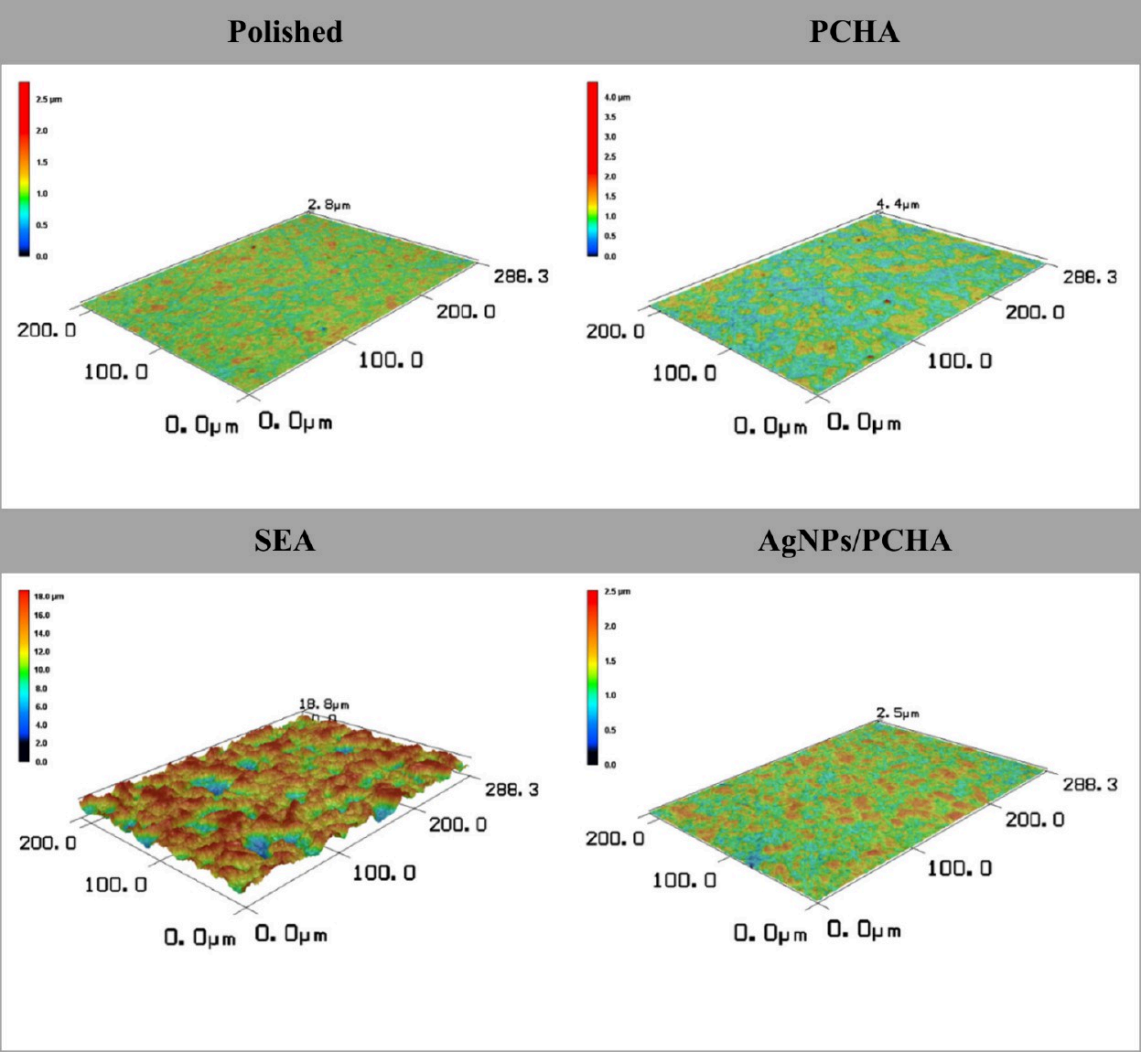
Three-dimensional surface reconstructions of titanium samples obtained by laser scanning confocal microscopy. The color scale represents surface height variations.

**Table 3:** Surface Roughness Parameters Ra, Rq, and Rp Are Presented as Median and Interquartile Range (In Parentheses)^a^

Group	Ra (μm)	Rq (μm)	Rp (μm)	Rv (μm)	Rsk	Rku
**Polished**	0.039 (0.010)a	0.047 (0.013)a	0.076 (0.026)a	0.11 ± 0.08a	–0.04 ± 0.03	2.44 ± 0.02
**SAE**	0.309 (0.021)b	0.373 (0.026)b	0.668 (0.088)b	0.59 ± 0.07b	0.17 ± 0.08	2.49 ± 0.07
**PCHA**	0.037 (0.008)a	0.044 (0.011)a	0.074 (0.018)a	0.07 ± 0.01a	0.01 ± 0.09	2.47 ± 0.09
**AgNPs/PCHA**	0.038 (0.013)a	0.046 (0.015)a	0.080 (0.028)a	0.07 ± 0.01a	0.02 ± 0.08	2.45 ± 0.08

a Differences were sought using Kruskal–Wallis followed by Dunn’s post hoc test with Bonferroni adjustment; Rv, Rsk, and Rku are presented as mean ± standard deviation. Differences were sought using one-way ANOVA followed by Tukey’s post hoc test. Different letters mean significant differences (*p* < 0.05).

### 3.6 Wettability and Surface Free Energy

Polished, PCHA, and AgNPs/PCHA discs have shown contact angles below 90° threshold, indicating hydrophilic surfaces with thermodynamically favorable interactions with polar substances (Figure 12). Conversely, SAE discs displayed predominantly hydrophobic behavior, with most contact angles exceeding 90° threshold. Some authors have reported that the hydrophobicity of rough surfaces is transient and may result from air entrapment within microscale pores, leading to a surface that resists spontaneous wetting. To overcome this limitation, additional treatment steps using alkaline solutions have been proposed in the literature to enhance surface hydrophilicity,^39^ particularly in commercially available treatments using sandblasting and double acid-etching, which aim to improve osseointegration by combining microroughness with optimized wettability for better interaction with bodily fluids.^40^ Although initially hydrophobic, these surfaces become fully wettable after the first immersion cycle during dynamic contact angle testing.^39^

**Figure 12:**
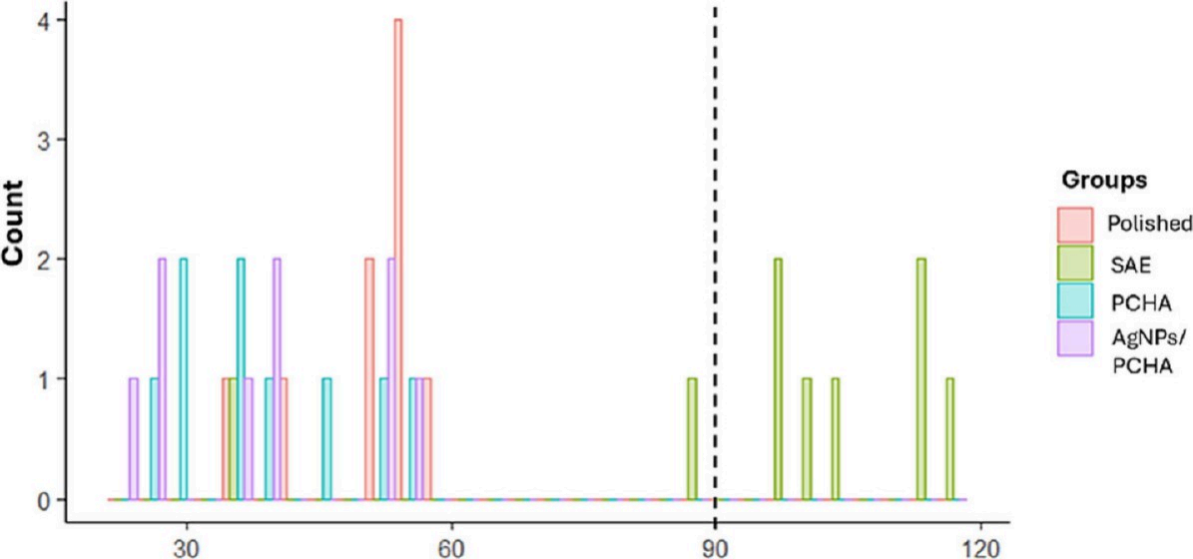
Contact angle distribution histogram of the Polished, SAE, PCHA, and AgNPs/PCHA discs. The dashed line indicates the 90° threshold separating hydrophilic (<90°) and hydrophobic (>90°) surface behavior.

Most metallic surfaces are inherently hydrophilic; however, when surface roughness is introduced, the interaction of energy with polar fluids may reduce, depending on surface chemistry and topography.^41^ This represents an ongoing challenge for the dental implant industry, to develop surfaces that are both hydrophilic and topographically rough. In this context, numerous studies have focused on the surface modification as a promising strategy to enhance the interaction of biomaterials with physiological fluids.^24,27,39,42^ The surface free energy recorded for our evaluated groups are displayed in Table 4.

**Table 4:** Mean Values of Contact Angles (Θ) And Surface Free Energy Parameters of the Tested Samples^a^

Group	Dispersive component	Polar component	Surface free energy	Contact angle (θ)
**Polished**	26.00 ± 2.84a	24.24 ± 4.85a	50.09 ± 5.10a	50.55
**SAE**	28.30 ± 3.65a	6.14 ± 14.61b	32.51 ± 9.15b	96.39
**PCHA**	20.10 ± 6.25a	35.90 ± 6.28c	56.39 ± 7.20a	38.66
**AgNPs/PCHA**	19.40 ± 9.29b	38.82 ± 10.32c	55.70 ± 6.08a	38.49

a Surface free energy and its polar and dispersive components were analyzed using linear regression. For the dispersive component, regression was conducted with robust standard error correction. Data are presented as mean ± standard deviation. Different letters indicate significant differences (*p* < 0.05).

The SAE discs exhibited a significantly lower total surface free energy compared to the other discs (*p* < 0.0001), whereas PCHA and AgNPs/PCHA maintained values comparable to or slightly higher than the Polished discs, indicating that the experimental coatings preserved or enhanced surface wettability. The influence of surface treatment on the surface free energy was supported by linear regression models (adjusted R^2^ = 0.648). With respect to the polar and dispersive components of surface energy, PCHA and AgNPs/PCHA showed significantly higher polar contributions compared to Polished (*p* < 0.001), which may favor interaction with polar biomolecules. Conversely, the SAE exhibited a significant reduction in the polar component (*p* = 0.002), which may be less favorable for initial protein adsorption. A significant reduction in the dispersive component was also observed for AgNPs/PCHA (*p* = 0.003), while SAE and PCHA remained comparable to the Polished discs. These results suggest that the presence of carbonated hydroxyapatite in the PCHA and AgNPs/PCHA coatings may be associated with an increase in the polar component of the surface, while no substantial changes in total surface energy were observed. In contrast, although the SAE treatment promoted the formation of TiOH_*x*_ groups, it resulted in reduced wettability and surface energy, probably due to its pronounced microroughness.

### 3.7 Film–Substrate Adhesion Test

The adhesion of the experimental coatings to the titanium surfaces occurs through electrostatic forces involving the charge difference between titanium dioxide and the negatively charged polar heads of the phospholipids.^54^ The adhesion strength of the experimental films to the titanium substrate was evaluated and the maximum force values (N) were displayed in Table 5.

**Table 5:** Mean and Standard Deviation (in Parentheses) Of Maximum Tensile Force^a^

Grupo	Maximum force (N)
**Polished**	2.05 (0.33)
**SAE**	2.07 (0.59)
**PCHA**	2.17 (0.24)
**AgNPs/PCHA**	2.12 (0.33)

a Data was analyzed using analysis of variance (ANOVA).

The mean maximum force values obtained from the adhesion tests for all samples showed no significant differences between the groups (p = 0.966), indicating that surface treatment variations did not have a substantial impact on adhesion strength. The statistical analysis revealed an F-value of 0.087, which suggests that the observed variation between the groups was negligible when compared to the within-group variation, further supporting the conclusion of no significant differences in adhesion performance. These findings suggest that the different surface treatments applied to the titanium substrate, including those that involved the formation of hybrid films in the experimental groups, did not significantly influence the adhesive properties of the coatings. Despite the different surface modifications, all coatings demonstrated similar and satisfactory adhesion to the titanium base material, highlighting the robustness and consistency of the adhesion performance across the various treatment conditions.

### 3.8 Cell Proliferation

Upon contact with bodily fluids, the implant surface undergoes dynamic changes driven by rapid ionic, molecular, and cellular interactions. Surface modifications, such as oxide films or bioactive coatings applied to commercially pure titanium or its alloys can modulate the cellular responses.^6^ All the surfaces tested in the MTT assay supported cellular proliferation over a 5-day period (Figure 13). As expected, Polished discs presented high osteoblast proliferation since they are characterized by low roughness and a titanium dioxide surface, which facilitate effective protein adsorption and cell adhesion.^31^ Increased roughness surface has been shown to enhance protein adsorption and cellular adhesion.^43^ However, in the present study, SAE discs presented the lower levels of osteoblast proliferation. A probable rationale may be attributed to the reduced wettability caused by oxygen entrapment within the pores of the rough surfaces. In this study, the experimental surfaces were modified with a biomimetic nanostructured interface composed of carbonated hydroxyapatite crystals synthesized *in situ* via wet chemical methods, with nucleation occurring on an organic matrix supersaturated with Ca^2+^. The AgNPs/PCHA surface additionally incorporated silver nanoparticles. Both surfaces exhibited increased hydrophilicity and, consequently, enhanced cell proliferation, probably due to increased surface polarity and selective protein adsorption.^31^ Noteworthy, surfaces functionalized with bioactive nanoparticulate ceramics have demonstrated enhanced osteoblast proliferation.^27^

**Figure 13:**
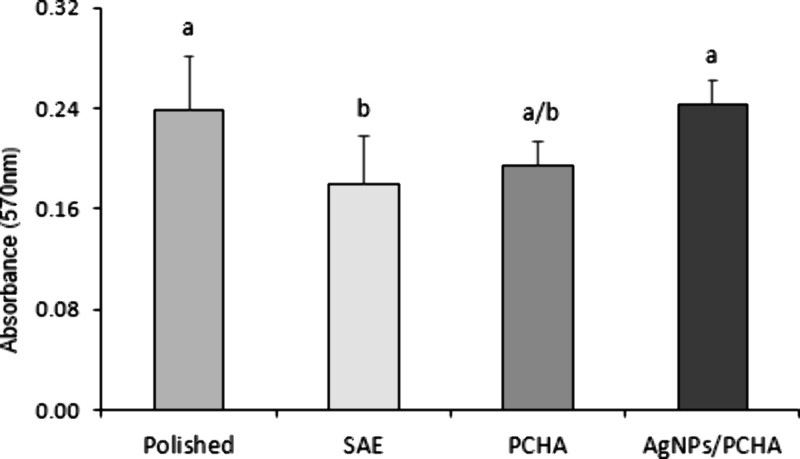
Bar chart showing the mean osteoblast proliferation on titanium discs after 5 days of culture. Data were analyzed using one-way ANOVA followed by Tukey’s post hoc test. Different letters indicate significant differences between groups (*p* < 0.012).

A slight delay in the cell proliferation was observed on the PCHA discs, possibly due to its elevated Ca^2+^ content, which can inhibit early stage osteoblast activity but typically allows recovery of proliferative capacity after 7 days.^24,31,37^ EDS analysis revealed a calcium concentration approximately three times higher in PCHA compared to AgNPs/PCHA, supporting this hypothesis. In the other hand, the higher incorporation of CO_3_^2–^ into the nonstoichiometric hydroxyapatite structure of AgNPs/PCHA may explain its superior proliferative performance, suggesting a cellular preference for spherical and nanostructured carbonated hydroxyapatite particles.^44^

Future studies should include complementary biological analyses such as long-term cytotoxicity, macrophage-mediated inflammatory response (cytokine release), and osteogenic differentiation markers (ALP activity and mineralization) to better assess the clinical potential of Ag-containing hybrid coatings.

### 3.9 Biofilm Morphology

Figure 14 represents the SEM images of the biofilm morphology on the titanium surfaces. In the Polished discs, the surface observed at 3000× magnification appeared smooth with minimal machining marks and no clear signs of bacterial adhesion. At 7500×, small surface defects were visible, yet no substantial biofilm formation was detected, suggesting limited bacterial colonization. At 10000×, the surface remained largely clean, with only isolated areas showing initial bacterial interaction. In this investigation, the smooth machined surfaces constituted a more difficult environment for bacterial adhesion and proliferation. The absence of valleys and hollow spaces could be a rationale for these findings, reducing bacteria trapping while facilitating the removal of planktonic bacteria by saliva flow.^45^

**Figure 14:**
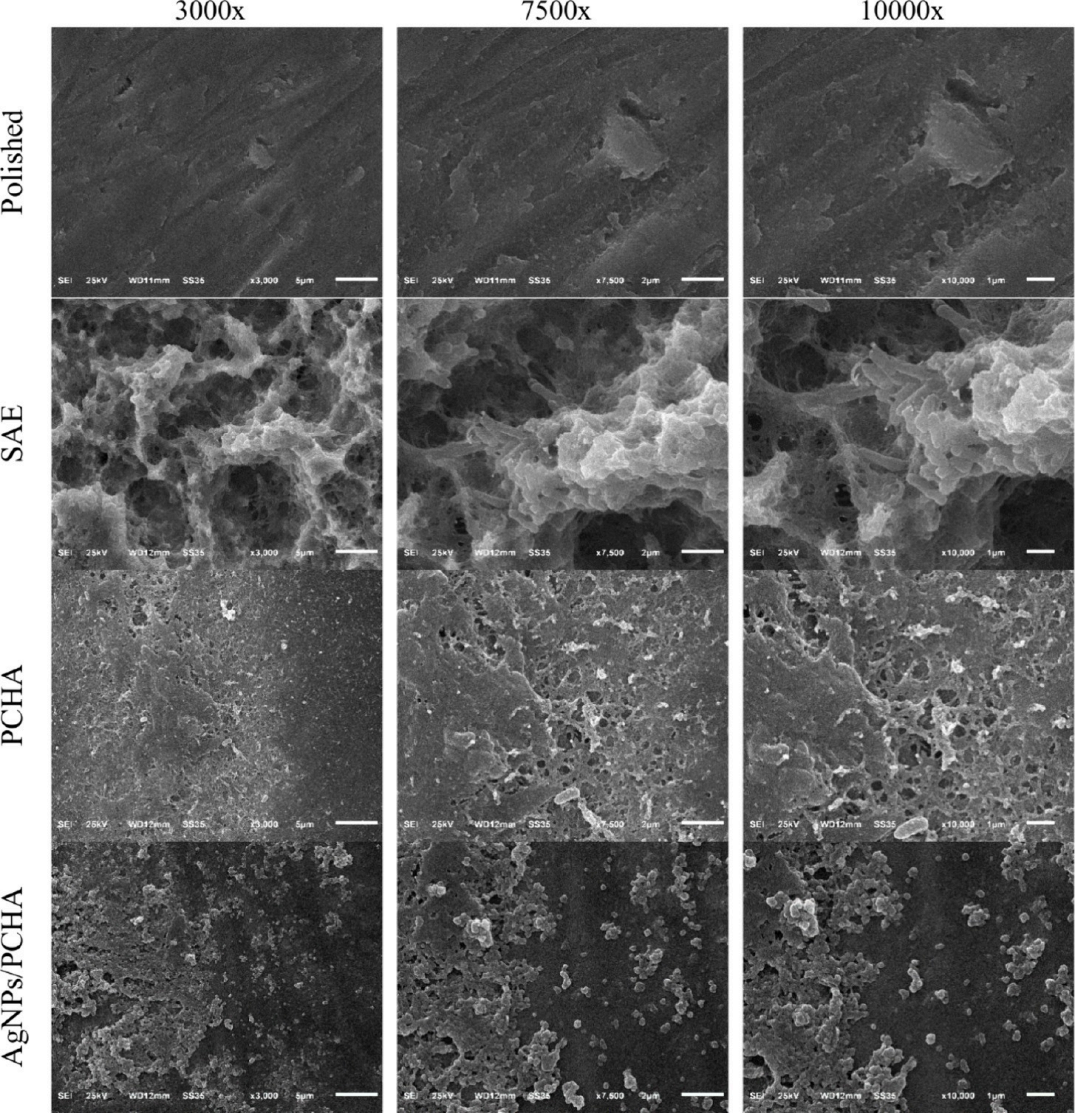
SEM images at three different magnifications showing the morphology of biofilm–surface interaction on titanium discs after 48 h of intraoral exposure in human volunteers.

In contrast, the SAE discs exhibited a markedly different profile; at 3000×, a dense and fibrous extracellular matrix was shown filling the micropores. At 7500×, a well-organized biofilm structure with multiple layers of extracellular polymeric substance (EPS) was evident, including visible pores and cavities typical of mature biofilms. At 10000×, bacterial cells were fully embedded within the EPS, indicating that the increased surface roughness enhanced bacterial retention by providing anchoring niches and protective microenvironments.

For the PCHA discs, early biofilm formation was observed at 3000×, with small bacterial clusters dispersed on the surface. At 7500×, moderate EPS production and localized bacterial aggregates were present. Extensive areas also remained exposed, reflecting a less dense and less organized biofilm. At 10000×, cells were embedded in a sparse EPS network, suggesting limited colonization.

In the AgNPs/PCHA discs, biofilm development was more evident than in PCHA, though still lower than in SAE. At 3000×, dispersed bacterial clusters were observed with some denser aggregations. At 7500× the biofilm lacked a well-structured network and at 10000× the surface remained partially uncovered, indicating localized and less mature biofilm formation. While bacterial adhesion occurred, biofilm density and organization were clearly inferior to those observed on the rough surface of the SAE discs. There is no consensus in the literature regarding the effect of topography (mainly roughness) on the bacterial adhesion.^46^

In this investigation, the experimental coatings have proved to reduce the biofilm formation. It is hypothesized that the CHA formed on the surface of PCHA and AgNPs/PCHA discs may reduce the bacterial adhesion by interfering with cell wall adhesins,^47^ although this effect remains to be experimentally verified. Future studies involving surface proteomics or adhesin binding assays could provide direct evidence for this proposed mechanism.

### 3.10 Biofilm Coverage Area and Cell Viability

Figure 15 illustrates the quantification of oral biofilm coverage on the titanium surfaces (n = 2) after 48 h of intraoral exposure. Data was provided as descriptive statistics. The groups exhibited distinct distributions regarding the percentage of surface area covered by viable and nonviable cells. SAE, PCHA, and AgNPs/PCHA discs tended to present higher percentages of viable cell coverage compared to Polished discs. SAE discs showed a concentration of higher coverage values, whereas Polished discs displayed a narrower distribution of coverage values, with most values clustered at lower coverage percentages. Regarding surface coverage by dead bacterial cells, Polished discs presented greater dispersion, suggesting that the smooth surface may limit bacterial adhesion under the conditions evaluated.

**Figure 15:**
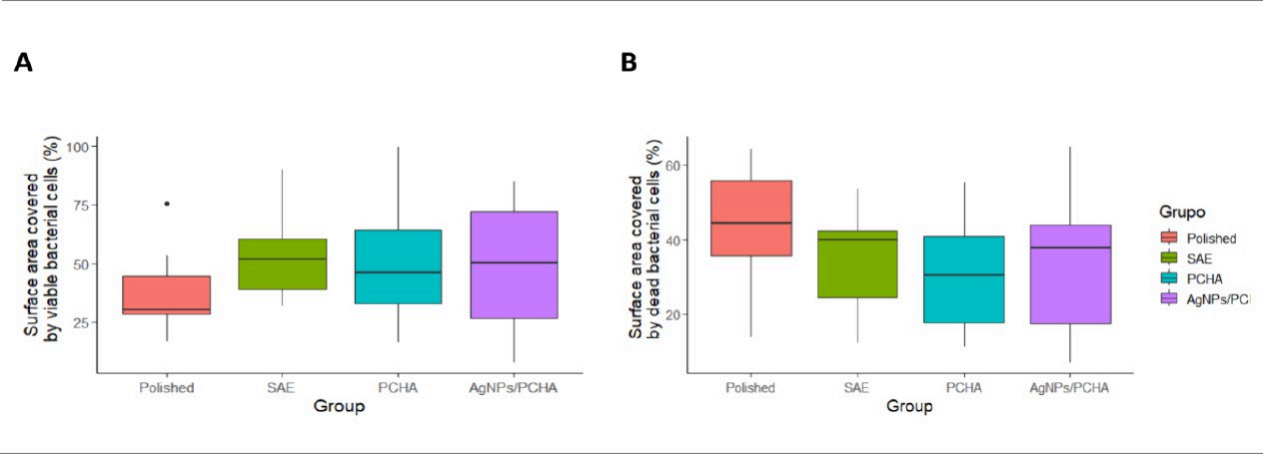
Boxplot displaying median, interquartile range, and total range of values of biofilm coverage on the discs. (A) Surface area covered by viable bacterial cells (%). (B) Surface area covered by dead bacterial cells (%).

On the surface of Polished discs, bacterial clusters were predominantly composed of nonviable cells (Figure 16). Smooth surfaces offer fewer mechanical anchorage points for microorganisms, which can impair bacterial attachment, particularly during the initial adhesion phase that requires close physical contact between the bacterial cell and the substrate. Consequently, the absence of microstructural features that promote niche formation may limit early biofilm establishment on polished surfaces.

**Figure 16:**
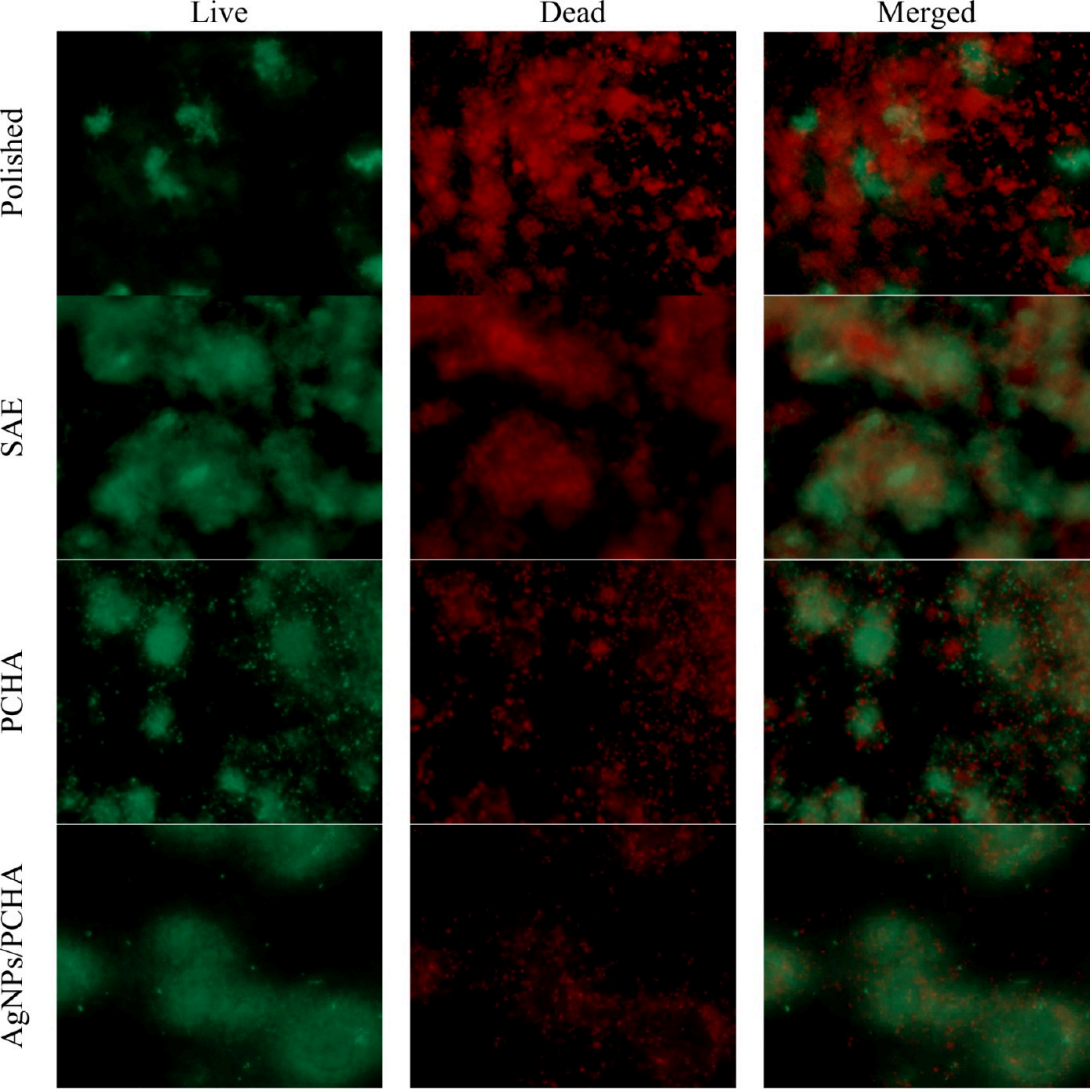
Fluorescence microscopy images of biofilms formed on the surface of titanium discs after 48 h of intraoral exposure. Viable cells appear green (SYTO 9 stain), while nonviable cells appear red (propidium iodide).

In contrast, the increased rough surface of the SAE discs exhibited denser bacterial aggregates, including both viable and nonviable cells. The presence of micropores increases the effective contact area for bacterial adhesion and may offer protection from fluid shear forces. These irregularities generate protected microenvironments with localized nutrient retention and reduced oxygen availability, potentially facilitating initial colonization.^48^ The submicrometer-engineered surfaces PCHA and AgNPs/PCHA were associated with a lower proportion of nonviable cells. In these groups, the biofilm exhibited a more dispersed distribution. The absence of microporosity on the hybrid-coated surfaces may have limited homogeneous biofilm expansion, resulting in a seemingly reduced effective colonization area.

### 3.11 Bacterial Biovolume

The analysis of bacterial biovolume on the different titanium surfaces revealed marked variations in both biofilm formation and viability (Table 6). The tridimensional reconstruction of the biofilm was presented in Figure 17. The Friedman test indicated a trend toward group differences in live biovolume (p = 0.066) and a significant difference in dead biovolume (p = 0.029). Post hoc analyses revealed a significant difference between SAE and the Polished surfaces for dead biovolume (p = 0.037).

**Figure 17:**
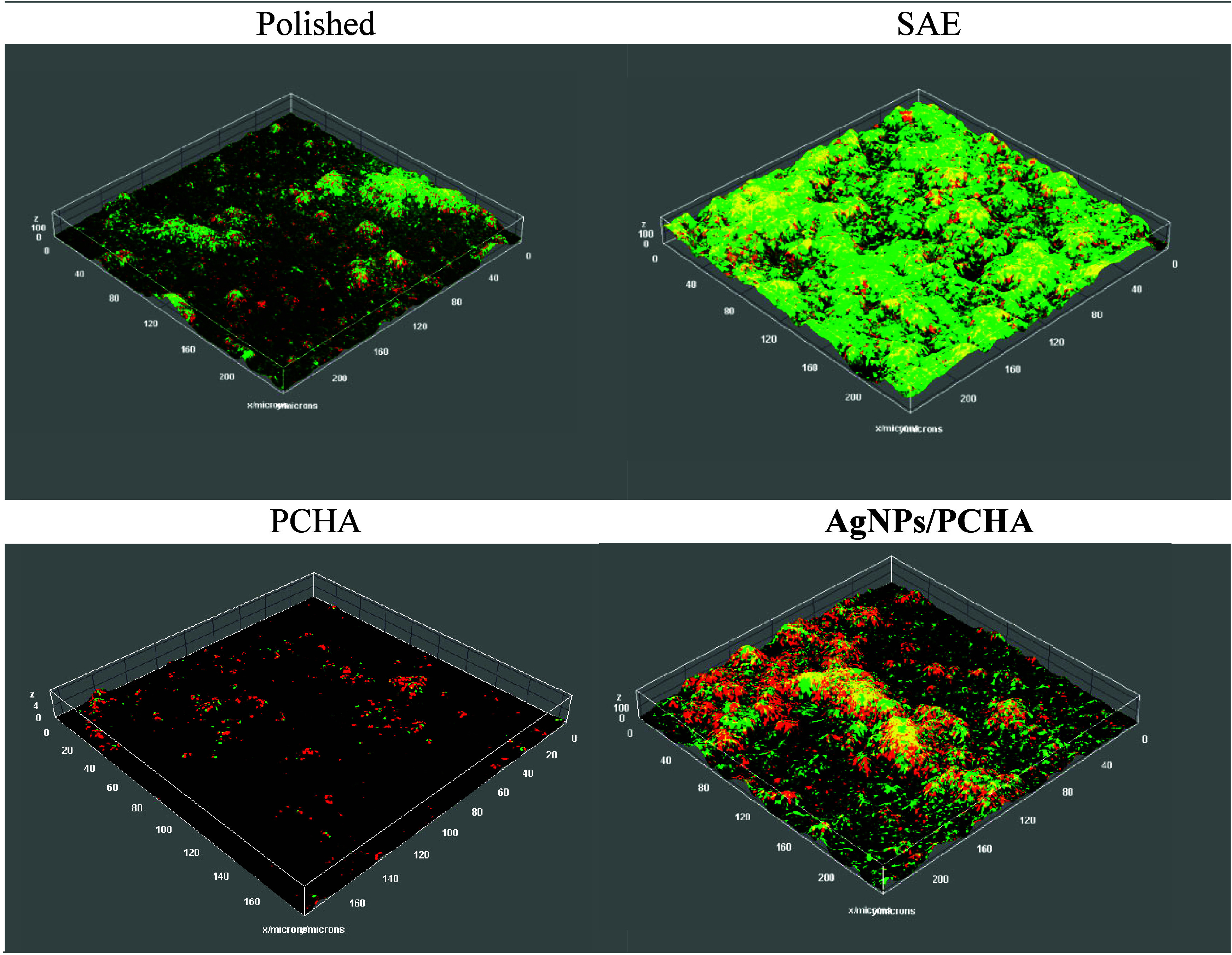
Tridimensional reconstruction of the biofilm generated by maximum intensity projection in ImageJ software, using the Interactive 3D Surface Plot plugin.

**Table 6:** Mean Values, Standard Deviation, and 95% Confidence Interval of Live and Dead Biofilm Biovolumes (×10^4^ μm^3^) Formed on the Titanium Surfaces

	Live Biovolume			Dead Biovolume		
Group	Mean	Standard Deviation	95% Confidence Interval (Lower–Upper)	Mean	Standard Deviation	95% Confidence Interval (Lower – Upper)
**Polished**	10.35	15.87	(4.42–16.28)	9.17	15.38	(3.47–14.91)
**SAE**	37.28	55.65	(16.5–58.06)	38.93	36.67	(25.23–52.62)
**PCHA**	4.34	5.00	(2.47–6.21)	6.06	6.14	(3.77–8.35)
**AgNPs/PCHA**	32.42	100.33	(5.04–69.88)	13.98	33.13	(1.61–26.35)

Generalized linear mixed models with Tweedie distribution confirmed significant increases in the live biovolume for SAE (β = 1.43; *p* < 0.001) and AgNPs/PCHA (β = 1.14; p = 0.0028), as well as in the dead biovolume for SAE (β = 1.70; *p* < 0.001) when compared to the Polished surfaces. The roughened SAE surfaces exhibited the highest biofilm accumulation with increased values for both viable (4.2-fold; *p* < 0.001) and nonviable biovolumes (5.5-fold; *p* < 0.001). This suggests that a more complex topography has acted in two ways: first, the microgaps have favored bacterial adhesion and colonization; afterward, they have reduced nutrient availability for bacteria to grow.

On the AgNPs/PCHA surface, viable biovolume increased selectively (3.1-fold; p = 0.0028), while nonviable cell levels remained unchanged. This behavior may reflect the low concentration of bioavailable silver (0.154 mg Ag·L^–1^) indicated by ICP-OES analysis, rather than an intrinsic lack of antimicrobial activity of the nanoparticles. Theoretical amount of Ag available in the LB subphase is provided in Supplementary S1. Under the experimental conditions of this study, the silver concentration did not reach the minimum inhibitory level required to produce a measurable antimicrobial effect of AgNPs. Further investigations are therefore needed to identify the optimal concentration and deposition conditions that provide sufficient silver bioavailability to achieve antimicrobial activity while maintaining biocompatibility. In contrast, the PCHA surface, coated with type A carbonated hydroxyapatite, displayed the lowest biofilm volume, predominantly composed of nonviable cells, indicative of passive antimicrobial effects associated with its nanostructure. Although statistical differences were not observed compared to the Polished surfaces, data suggests that this surface may have potential in preventing early microbial adhesion. These findings underscore that the topographic and chemical modulation of implant surfaces can influence not only the extent of biofilm development but also its viability. This opens promising avenues for designing implant materials that balance desired cellular adhesion with biofilm control.

### 3.12 Checkerboard DNA–DNA Hybridization Quantification

The total bacterial count recovered from the investigated groups was illustrated in Figure 18. Significant differences were found between different tested surfaces (Friedman; *p* = 1.34 × 10^–5^). Pairwise multiple comparisons revealed the following results: Polished - SAE (*p* = 0.040), Polished – PCHA (*p* = 0.032), Polished - AgNPs/PCHA (*p* = 0.210), SAE - PCHA (*p* = 1. 60 × 10^–5^), SAE - AgNPs/PCHA (*p* = 0.0004), PCHA - AgNPs/PCHA (*p* = 0.232). These findings corroborate with the results observed in the bacterial biovolume and biofilm formation, in which PCHA and PCHA/AgNPs surfaces were shown to be effective in reducing biofilm formation. The experimental coatings presented reduced values of bacterial load, similar to those found for Polished surfaces. The lower total bacterial count was recorded for PCHA surfaces, confirming that both roughness and carbonate hydroxyapatite played a relevant role in the biofilm formation.^46,47^

**Figure 18:**
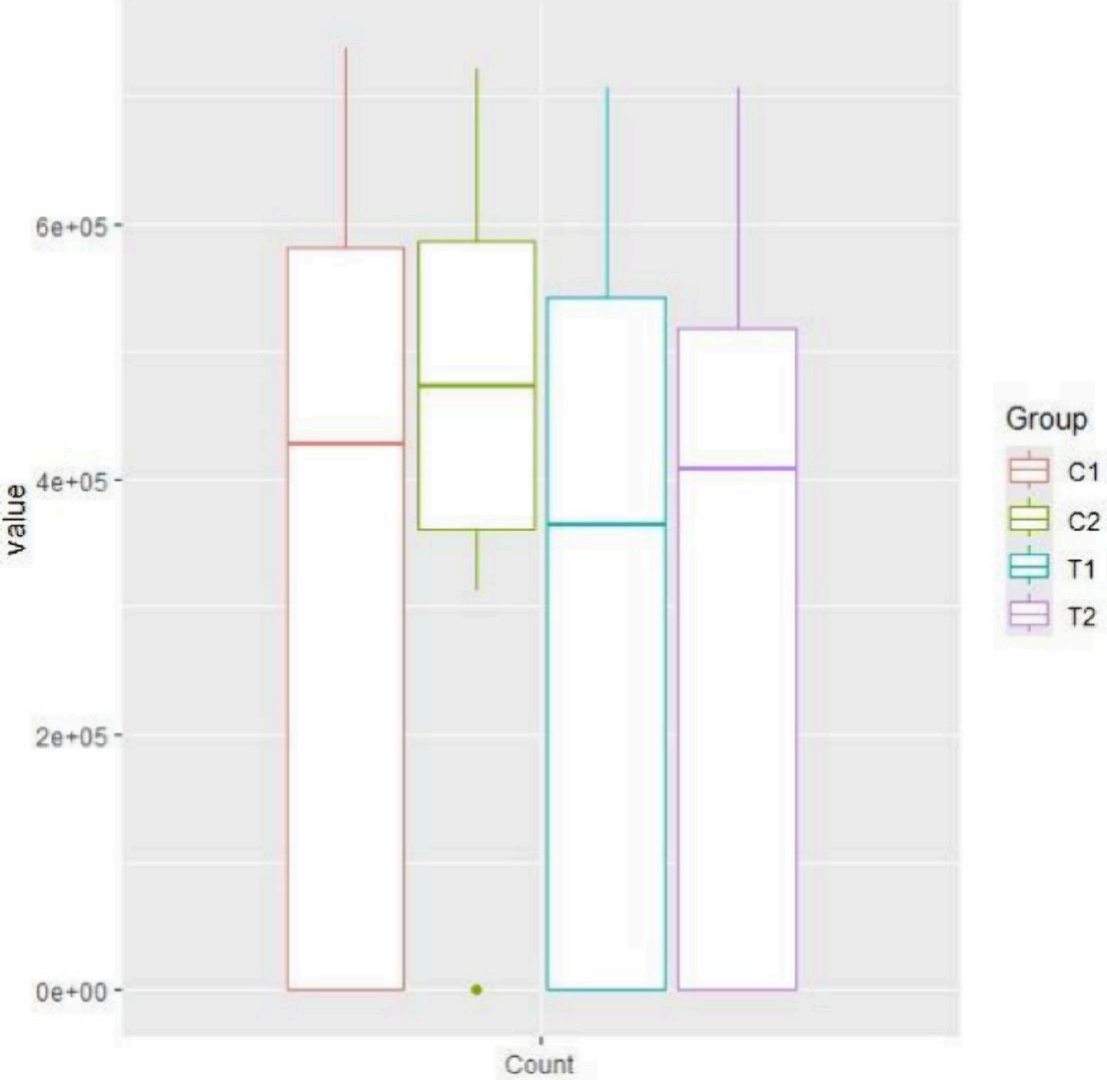
Median, maximum, minimum, and interquartile range of total bacterial count from tested surfaces. Differences sought by Friedman followed by pairwise comparisons using Wilcoxon rank sum corrected by Bonferroni (*p* < 0.05). C1: Polished; C2: SAE; T1: PCHA; T2: AgNPs/PCHA.

Figure 19 illustrates the individual bacterial count recovered from investigated surfaces. A generalized linear mixed model (GLMM) with a negative binomial distribution (nbinom2) and a log-link function were employed to compare the individual bacterial counts across surface groups, accounting for subject-level random effects to control the intraindividual correlation. Overall, the experimental surfaces (PCHA and AgNPs/PCHA) presented the lower levels of target species, except for *S. mitis*, *P. melaninogenica* and *K. pneumoniae*. Klebsiellla are opportunistic pathogens having an increased association with periodontal pockets. They can be highly virulent and resistant to multiple antibiotics.^49^ Of the utmost importance, both experimental coatings have substantially reduced the levels of *T. denticola* and *P. gingivalis*, which display strong synergy in the formation of polymicrobial biofilms leading to increased biovolume. These species belong to the red complex of periodontal diseases and are closely associated with the etiology of peri-implantitis.^50^ Streptococcus genera, related to the early stage of biofilm formation, and other relevant species associated with the biofilm maturation were found in significant reduced counts for PCHA and AgNPs/PCHA when compared to SAE surfaces.

**Figure 19:**
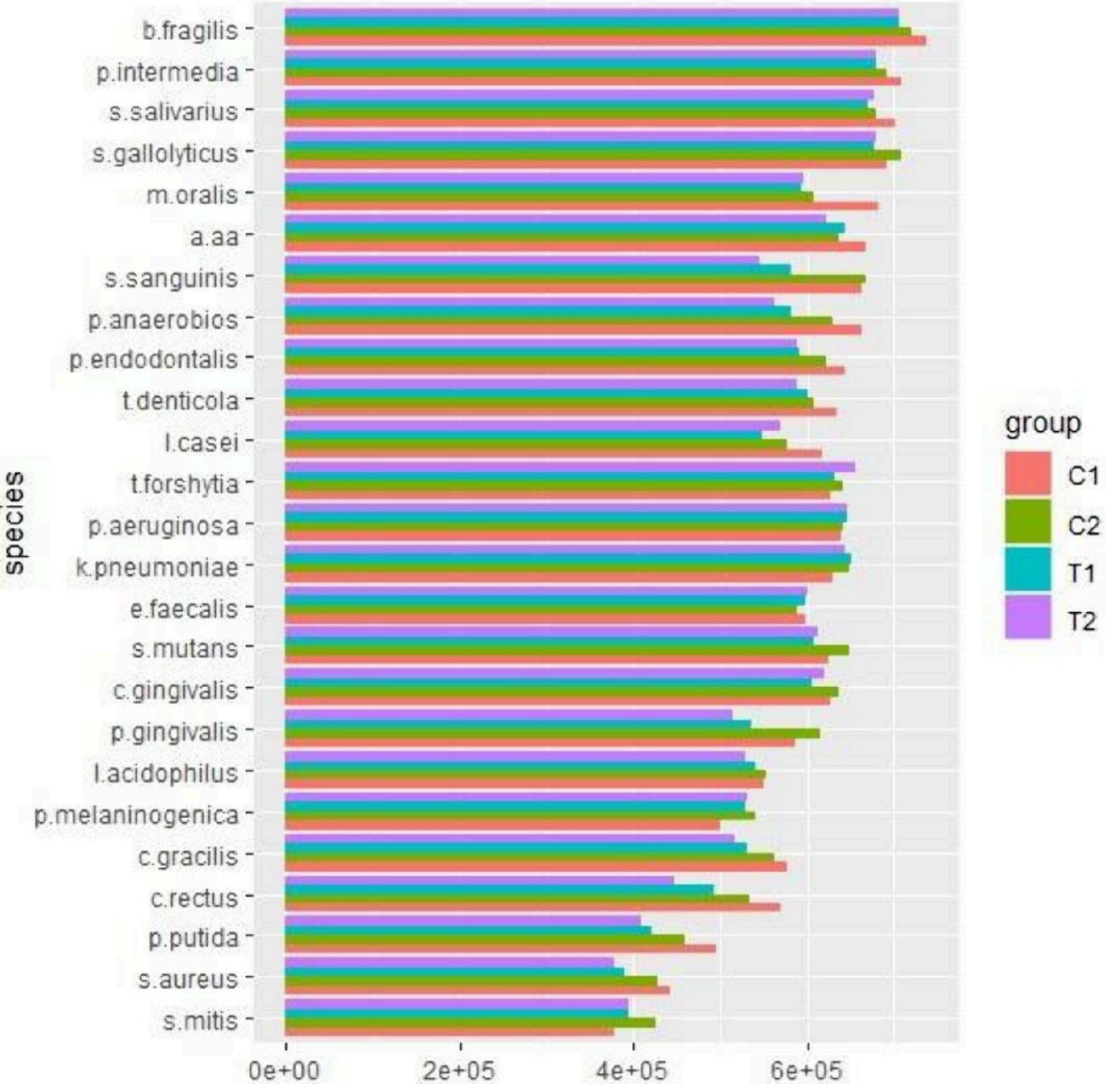
Individual bacterial count (Mean, ×10^5^) from tested surfaces. C1: Polished; C2: SAE; T1: PCHA; T2: AgNPs/PCHA.

The estimated levels of target species suggest meaningful biological trends, particularly with the higher bacterial colonization observed in SAE and the reduced counts in experimental coatings. The differences in the microbial profile found for SAE and experimental coatings may be supported by the physicochemical interactions between bacteria and surfaces. Rough surfaces accumulate and retain more bacteria, facilitating the microbial adhesion and attachment. In addition, bacteria with high surface free energy preferentially adhere to substrates with high surface free energy. Considering that most of oral bacteria have high surface energy, we might conclude that the higher levels of species found for SAE were related to its high roughness and surface free energy.^51^

Figure 20 shows the prevalence (%) of target species across the samples. Most species exhibited a prevalence above 75%. *P. intermedia* and *A. actinomycetemcomitans* were the most prevalent, while *L. acidophilus* and *M. oralis* were the least prevalent. The microbial community was diverse and relatively evenly distributed among samples. This widespread presence of target species was expected since a diverse and complex community structure harboring numerous and diverse commensals and opportunistic microorganisms has been reported in healthy individuals.^52,53,55^

**Figure 20:**
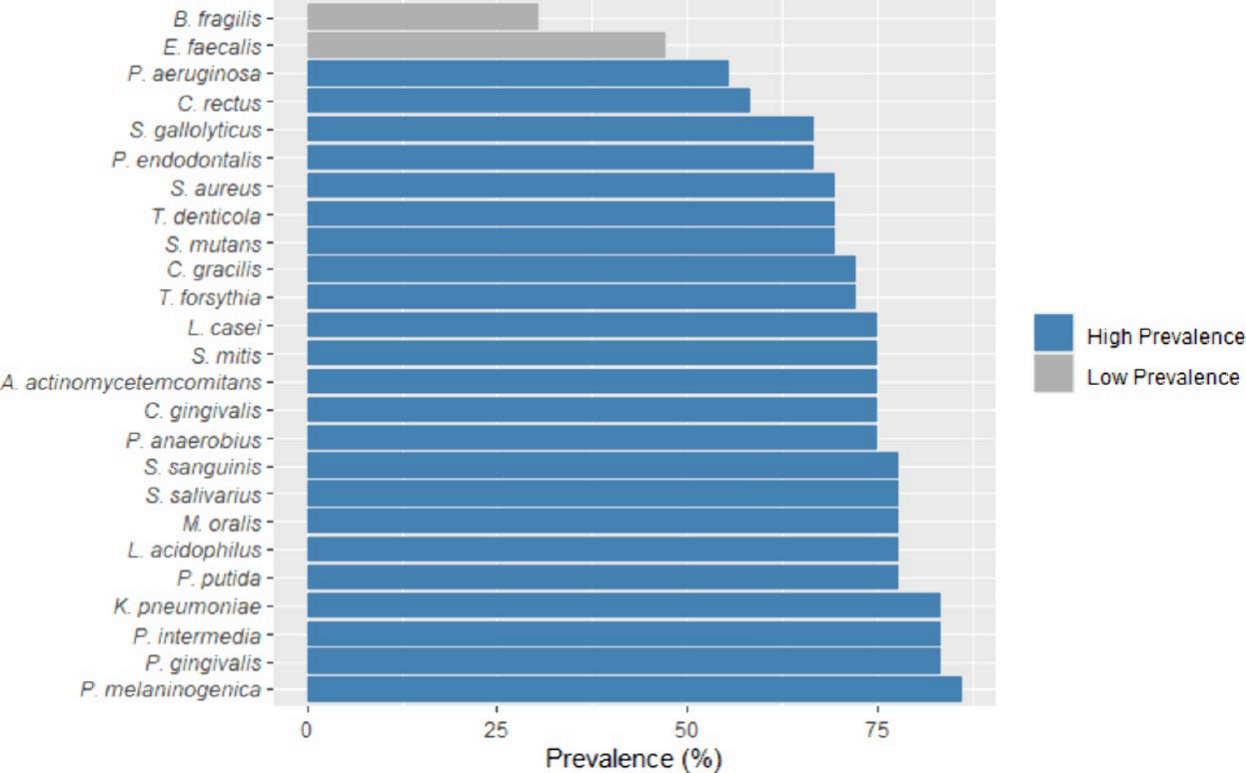
Prevalence (%) of bacterial species across the samples.

### 3.14 Limitations of the Current Research

Some limitations should be considered when interpreting the results of this study. First, the relatively short exposure time of the intraoral devices (48 h) limits the ability to draw long-term conclusions regarding the clinical performance of the experimental surfaces. While this duration was adequate to meet the objectives of the current study, longer exposure times would be necessary to evaluate the surfaces’ durability and effectiveness under more clinically relevant conditions. Additionally, the sample size, although sufficient for the present analysis, could be expanded to improve the representativeness of the findings and enhance the statistical power of the results. Future research should focus on extending the exposure period to better assess the surfaces’ potential for osteoinduction and biofilm regulation over time. Moreover, complementary analyses are recommended to further characterize the materials’ properties, including transmission electron microscopy (TEM) to investigate the ultrastructure and integrity of the hybrid films. Mechanical stability tests, such as wear and durability assessments under fluid exposure, will also be essential for evaluating the robustness and long-term applicability of these surfaces in clinical settings.

## 4 Conclusions

Despite the recent advances in the titanium surface modifications, the bacterial adhesion and colonization of implant-related sites still lead to inflammatory reactions and remains as a major challenge in the oral biofilm controlling. Surface modifications are usually performed to enhance antibacterial activity and osteogenic capability. In this study, PCHA and AgNPs/PCHA coatings were developed to promote antibacterial and antibiofilm activities while maintaining the osteogenic capability. Both experimental coatings were shown as a stable hybrid film formed over the TiO_2_ with a dense layer of carbonated hydroxyapatite, resulting in less roughness and hydrophilic surfaces. The results showed that PCHA and AgNPs/PCHA coatings not only played a relevant role in reducing the oral biofilm formation but also presented bacterial adhesion and proliferation reduction ability. They may also be beneficial for promotion of osteogenesis. Meanwhile, the incorporation of silver nanoparticles into the hybrid film did not result in enhanced antibacterial performance, likely due to the low levels of bioavailable silver, as confirmed by ICP-OES analysis.

## Supplementary Material



## Notes

Theoretical Maximum Concentration of Silver (Ag) in the LB subphase; Theoretical Maximum Concentration of Boron (B) in the LB subphase; Determination of Ag by Inductively Coupled Plasma Optical Emission Spectrometry. (PDF)
