# Supplementary material for: Antibacterial and Antibiofilm Activity of Titanium Treated with Hybrid Phospholipid Films Containing Carbonate Hydroxyapatite and Silver Nanoparticles
Source: ACS Omega. 2026 Feb 26;11(9):14479–500. doi: 10.1021/acsomega.5c09762 (PMC12980427; doi:10.1021/acsomega.5c09762)
Supplement: Supplementary file 1 [file ao5c09762_si_001.pdf]

Antibacterial and antibiofilm activity of titanium treated with hybrid phospholipid films containing carbonate hydroxyapatite and silver nanoparticles

Carla Roberta de Oliveira Maciel[1], Ailton Cravo Moraes Filho[1], Antonieta Catalina Varela Garcia[1], Viviane de Cássia Oliveira[1], Ana Paula Ramos[2], Ricardo Faria Ribeiro[1], Marcelle Beathriz Fernandes da Silva3, Rafael Soares Stenico[2], Marcia Andreia Mesquita Silva da Veiga[2], Cássio do Nascimento[1*]

[1]Ribeirão Preto School of Dentistry, Department of Dental Materials and Prosthodontics, University of São Paulo, Ribeirão Preto 14040-904, São Paulo, Brazil;
[2]Faculty of Philosophy, Sciences and Letters of Ribeirão Preto, Chemistry Department, University of São Paulo, Ribeirão Preto, 14040-900, São Paulo, Brazil;
[3]Bone Research Lab, Ribeirão Preto School of Dentistry, University of São Paulo, Ribeirão Preto, São Paulo, Brazil.
*Corresponding author: e-mail: cassionasc@forp.usp.br Phone: +55 16 3315-4095

Supporting Information

Supplementary S1. Theoretical Maximum Concentration of Silver (Ag) in the LB subphase.

Initial data (from the experimental protocol)

- $AgNO_3$ solution: 1.0 mmol·L^{-1} .
- Volume of this solution used for the synthesis: 10.0 mL = 0.0100 L.
- After the reaction, 40.0 mL = 0.0400 L of $NaBH_4$ was added, resulting in a total colloid volume of approximately 50.0 mL = 0.0500 L.

- Molar mass of silver (Ag): 107.868 g·mol^{-1}.
- For LB deposition, 1.0 mL = 0.0010 L of this colloid was used, which was added to a total subphase volume of 140 mL = 0.1400 L (139 mL + 1 mL).

Moles of Ag originally present in the synthesis flask:

$$N_{Ag} = 1 \text{ mmol.L}^{-1} \times 0.0100 \text{ L} = 1.0 \times 10^{-5} \text{ mol}$$

Colloid concentration after mixing (assuming total volume = 0.0500 L). Concentration = moles / volume:

$$C_{colloid} = 1.0 \times 10^{-5} \text{ mol} / 0.0500 \text{ L}$$

$$C_{colloid} = 2.0 \times 10^{-4} \text{ mol.L}^{-1}$$

Moles / mass in the 1.0 mL aliquot used in the subphase:

N_{1mL} = 2.0 x 10^{-4} mol.L^{-1} x 0.0010 L = 2.0 x 10^{-7} mol

Theoretical final concentration in the subphase (after dilution in 140 mL = 0.1400 L). Subphase concentration = moles in the aliquot / subphase volume:

$C_{subphase}$ = 2.0 x 10^{-7} mol / 0.1400 L = 1.4285714 x 10^{-6} mol.L^{-1}

$C_{subphase =}$ 1.4285714 x 10^{-6} mol.L^{-1} x 107.868 g.mol^{-1} = 0.154 mg.L^{-1}

Supplementary S2. Theoretical Maximum Concentration of Boron (B) in the LB subphase.

Initial data (from the experimental protocol)

- Synthesis: 10.0 mL of 1.0 mmol·L^{-1} $AgNO_3$ + 40.0 mL of 2.0 mmol·L^{-1} $NaBH_4$ → 50 mL suspension.

- Moles of Ag^+ added: 10.0 mL × 1.0 mmol·L^{-1} = 1.00 × 10^{-5} mol Ag
- Moles of $NaBH_4$ added: 40.0 mL × 2.0 mmol·L^{-1} = 8.00 × 10^{-5} mol BH_4^-
- It was considered that each BH_4^- ion contains 1 B atom.
- It was assumed that a simple stoichiometric reaction in which 1 mol of BH_4^- was consumed per 1 mol of Ag^+ (i.e., all Ag^+ is reduced and consumes 1 mol of BH_4^- per mol of Ag). This provides a maximum estimate of residual BH_4^- . In practice, part of the BH_4^- may also be consumed by hydrolysis or oxidation, so the actual value may be lower.

Residual moles of BH_4^- (assuming 1:1 consumption with Ag):

8.00 × 10^{-5} − 1.00 × 10^{-5} = 7.00 × 10^{-5} mol of BH_4^- remaining in the flask

Concentration of BH_4^- in the suspension:

C_B = 7.00 × 10^{-5} mol / 0.0500 L = 1.40 × 10^{-3} mol·L^{-1} = 1.4 mmol·L^{-1} (BH_4^-)

Conversion to mg·L^{-1} (atomic mass of B = 10.81 g·mol^{-1}):

1.40 × 10^{-3} mol·L^{-1} × 10.81 g·mol^{-1} = 0.015134 g·L^{-1} = 15.13 mg·L^{-1}

Theoretical concentration of B in the LB subphase after dilution (1 mL in the total subphase volume of 140 mL):

Dilution factor = 1 / 140 = 0.0071429

Subphase concentration ($mg·L^{-1}$) = 15.13 $mg·L^{-1}$ × 0.0071429 = ≈ 0.1081 $mg·L^{-1}$

Mass of B in 1.0 mL of the suspension (the aliquot added to the subphase)

15,13 $mg·L^{-1}$ / 1000 mL = 0,01513 mg = 15,13 µg of B in the 1 mL aliquot

Supplementary S3. Determination of Ag by Inductively Coupled Plasma Optical Emission Spectrometry.

Instrumental Parameters:

- Radiofrequency (W): 1500
- Axial Configuration
- Sample Flow Rate ($mL\ min^{-1}$): 1.0
- Plasma Gas Flow Rate ($L\ min^{-1}$): 8.0
- Auxiliary Gas Flow Rate ($L\ min^{-1}$): 0.2
- Nebulizer Gas Flow Rate ($L\ min^{-1}$): 0.7

Analytical Parameters:

- Analyte: Silver (Ag)
- Wavelength (nm): 328.068
- Slope of the Regression ($kg\ mg^{-1}$): 1,558,869.48
- Intercept of the Regression: −38,016.1266
- Working Range ($mg\ kg^{-1}$): 0.1 – 1.0
- Coefficient of Determination (R^2): 0.9983
- Pearson Correlation Coefficient (R): 0.9991
- Limit of Detection (LoD) ($mg\ kg^{-1}$): 0.03
- Limit of Quantification (LoQ) ($mg\ kg^{-1}$): 0.1
- Relative Standard Deviation (RSD) (%): 3.2

Silver Determination:

Table S1. Determination of silver ion concentration (mg/kg) by Inductively Coupled Plasma Optical Emission Spectrometry.

Sample	Silver Concentration (mg/kg)	Uncertainty (mg/kg)

**24 h – Disk 1**	<LoD	–
**24 h – Disk 2**	<LoD	–
**24 h – Disk 3**	<LoD	–
**48 h – Disk 1**	<LoD	–
**48 h – Disk 2**	<LoD	–
**48 h – Disk 3**	<LoD	–

Values below the limit of detection (LoD) do not allow confirmation of the presence of the element in the solution
